# Disagreement concerning atopic dermatitis subtypes between an English prospective cohort (ALSPAC) and linked electronic health records

**DOI:** 10.1093/ced/llae196

**Published:** 2024-05-16

**Authors:** Julian Matthewman, Amy Mulick, Nick Dand, Daniel Major-Smith, Alasdair Henderson, Neil Pearce, Spiros Denaxas, Rita Iskandar, Amanda Roberts, Rosie P Cornish, Sara J Brown, Lavinia Paternoster, Sinéad M Langan

**Affiliations:** London School of Hygiene & Tropical Medicine, London, UK; London School of Hygiene & Tropical Medicine, London, UK; Department of Medical and Molecular Genetics, School of Basic & Medical Biosciences, King’s College London, London, UK; Population Health Sciences, Bristol Medical School, University of Bristol, Bristol, UK; London School of Hygiene & Tropical Medicine, London, UK; London School of Hygiene & Tropical Medicine, London, UK; Institute of Health Informatics, UCL, London, UK; NIHR UCLH BRC, London, UK; BHF Data Science Centre, HDR UK, London, UK; London School of Hygiene & Tropical Medicine, London, UK; Independent Patient Partner; Population Health Sciences, Bristol Medical School, University of Bristol, Bristol, UK; MRC Integrative Epidemiology Unit, University of Bristol, Bristol, UK; Centre for Genomic and Experimental Medicine, Institute of Genetics and Cancer, University of Edinburgh, Edinburgh, UK; MRC Integrative Epidemiology Unit, Bristol Medical School, University of Bristol, Bristol, UK; NIHR Bristol Biomedical Research Centre, University Hospitals Bristol and Weston NHS Foundation Trust and University of Bristol, Bristol, UK; London School of Hygiene & Tropical Medicine, London, UK

## Abstract

**Background:**

Subtypes of atopic dermatitis (AD) have been derived from the Avon Longitudinal Study of Parents and Children (ALSPAC) based on the presence and severity of symptoms reported in questionnaires (severe–frequent, moderate–frequent, moderate–declining, mild–intermittent, unaffected–rare). Good agreement between ALSPAC and linked electronic health records (EHRs) would increase trust in the clinical validity of these subtypes and allow inference of subtypes from EHRs alone, which would enable their study in large primary care databases.

**Objectives:**

Firstly, to explore whether the presence and number of AD records in EHRs agree with AD symptom and severity reports from ALSPAC. Secondly, to explore whether EHRs agree with ALSPAC-derived AD subtypes. Thirdly, to construct models to classify ALSPAC-derived AD subtypes using EHRs.

**Methods:**

We used data from the ALSPAC prospective cohort study from 11 timepoints until age 14 years (1991–2008), linked to local general practice EHRs. We assessed how far ALSPAC questionnaire responses and derived subtypes agreed with AD as established in EHRs using different AD definitions (e.g. diagnosis and/or prescription) and other AD-related records. We classified AD subtypes using EHRs, fitting multinomial logistic regression models, tuning hyperparameters and evaluating performance in the testing set [receiver operating characteristic (ROC) area under the curve (AUC), accuracy, sensitivity and specificity].

**Results:**

Overall, 8828 individuals out of a total 13 898 had been assigned an AD subtype and also had linked EHRs. The number of AD-related codes in EHRs generally increased with the severity of the AD subtype. However, not all patients with the severe–frequent subtype had AD in EHRs, and many with the unaffected–rare subtype did have AD in EHRs. When predicting the ALSPAC AD subtype using EHRs, the best tuned model had an ROC AUC of 0.65, a sensitivity of 0.29 and a specificity of 0.83 (both macro-averaged). When different sets of predictors were used, individuals with missing EHR coverage were excluded, and subtypes were combined, sensitivity was not considerably improved.

**Conclusions:**

ALSPAC and EHRs disagreed not only on AD subtypes, but also on whether children had AD or not. Researchers should be aware that individuals considered to have AD in one source may not be considered to have AD in another.

## Teaser

Data from a prospective cohort (ALSPAC) on childhood atopic dermatitis (AD) subtypes, and data from the same individuals’ linked electronic health records showed poor agreement. Researchers should be aware that individuals considered to have AD in one source may not be considered to have AD in another.

**What is already known about this topic?**

- Childhood atopic dermatitis (AD) subtypes based on timing and severity of symptom reports were identified in prospective cohort data (ALSPAC).
- It is not understood whether these data agree with what is seen in electronic health records (EHRs) in AD.

**What does this study add?**

- Our findings indicate some correlation between ALSPAC symptom and severity reports, and the presence and number of AD-related EHRs.
- Between individuals with different ALSPAC subtypes, including the unaffected subtype, there was considerable overlap concerning their AD-related EHRs.
- These inconsistencies not only make it difficult to classify individuals’ ALSPAC subtypes using EHRs, but also suggest that the two data sources often do not agree on whether an individual has AD or not.

## Introduction

Atopic dermatitis (AD) is a common itchy skin disease with a high global burden in morbidity and healthcare costs.^1^ Four well-defined and recognizable subtypes (or phenotypes) of AD severity trajectories have been derived in the Avon Longitudinal Study of Parents and Children (ALSPAC). Latent class analysis is available using information on AD symptom presence and severity from questionnaires at 11 ages between 6 and 166 months (13.8 years). The subtypes are severe–frequent (*n* = 230, 3.9% of the development cohort), moderate–frequent (*n* = 408, 6.9%), moderate–declining (*n* = 676, 11%), mild–intermittent (*n* = 684, 12%) and unaffected–rare (*n* = 3929, 66%).^2^

Ideally, in the same people, AD-related electronic health records (EHRs) would indicate similar timing and severity of AD compared with data in ALSPAC. Such correlation could increase trust in the clinical validity of measures from both data sources. For example, if a parent had reported a severe rash in ALSPAC and there were AD-related records from the general practitioner (GP) diagnosing AD and prescribing an AD treatment in the same year, we could more easily trust that the child truly had severe symptoms of AD that year. However, previous studies have shown that longitudinal cohorts (like ALSPAC) do not always agree with EHRs.^3,4^

Besides assessing agreement, linkage between prospective cohorts, such as ALSPAC and EHRs, can potentially be used to enhance one data source using information from the other. If, using linkage, we could establish ways to determine children’s AD severity trajectories using EHRs alone, we could capitalize on the advantages of EHRs, such as larger sample sizes compared with prospectively collected cohorts such as ALSPAC. Current studies on AD in EHRs usually define AD as a single yes/no variable,^5^ where individuals with different subtypes are grouped together. This might result in inadequately broad recommendations or risk assessments. For example, development of a food allergy is a common comorbidity of AD, but the risk of this may vary depending on the AD subtype.

Here, we first explored agreement concerning AD between EHRs and ALSPAC cohort symptom and severity reports. Next, we studied the ALSPAC subtypes and developed and internally validated prediction models. ALSPAC-derived AD subtypes were used as the outcome to classify AD subtypes using linked EHRs.

## Methods

### Participants and data sources

ALSPAC originally enrolled 14 541 pregnant women with expected dates of delivery between 1 April 1991 and 31 December 1992 living in Avon, UK. The catchment area included the city of Bristol and surrounding urban and rural areas. There were 14 062 live births and 13 988 children who were alive at 1 year of age. Overall, 96% of participants were of White ethnicity. More details are provided at the study website (http://www.bristol.ac.uk/alspac/researchers/access). Resources include a data dictionary and a variable search tool. The linked cohort profile includes a description of the selection process and discussion of potential participation biases.^6–8^ We had access to data of 13 898 children in the core phase of ALSPAC, of whom we included individuals with both information on AD subtype and linked EHR data.

Via a postal campaign, ALSPAC formally sought to re-enrol study participants upon reaching adulthood, simultaneously seeking opt-out permission for linkage with EHRs. Then, linkage to anonymized local GP data (EHRs) was carried out for nearly 12 000 participants. This involved the transfer of both ALSPAC data and deidentified coded EHRs to a secure setting and linkage using a previously described ‘split file’ method.^9^

For some participants, linkage could only be established for parts of the study period, for example, if participants moved out of the area or to another practice outside the EMIS patient record system. For details, see ‘Linkage to GP records’ in the supplement from Cornish *et al.*^10^ For our study, we extracted records from EHRs using Read (version 2) codes and dictionary of medicines and devices (dm + d) product codes that were present in any of the prespecified code lists (Tables S1–3; see Supporting Information).^11^

### Variables from ALSPAC

#### ALSPAC questionnaire responses

Variables were analysed from ALSPAC questionnaire responses at ages 6, 18, 30, 42, 57, 69, 81, 103, 128, 140 and 166 months. These included presence of AD symptoms (questions on flexural rash, e.g. ‘child had rash in joints & creases in the past year’) and the severity of these AD symptoms (e.g. ‘severity of child’s itchy dry, skin rash’, with answers ‘no problem’, ‘mild’, ‘quite bad’ or ‘very bad’) (Table S4; see Supporting Information).

In secondary analyses, we defined parent-reported doctor’s AD and asthma diagnosis using the response to the question in ALSPAC on whether a doctor had ever diagnosed asthma or eczema by 166 months (‘Has a doctor ever actually said that he/she has asthma or eczema?’).

#### ALSPAC subtypes

We used the subtypes derived from ALSPAC from children in both the development and validation cohorts. In sensitivity analyses, we combined categories of the original subtypes.

### Variables from electronic health records

#### Timepoint-specific variables indicating atopic dermatitis

For each of the 11 timepoints in ALSPAC, using data from the 12 months prior to the respective timepoint, we assessed whether an individual had (i) an AD diagnosis, (ii) either an AD diagnosis or treatment (emollients, oral corticosteroids, systemic immunosuppressants, topical calcineurin inhibitors or topical corticosteroids), or (iii) both an AD diagnosis and a treatment in EHRs.

#### Variables derived from the entire follow-up period

From EHRs, we extracted information on diagnoses and treatments related to the severity and trajectory of AD. This information was judged by the study team, which included dermatologists and researchers with experience in AD research. We included allergic rhinitis, asthma-related records, asthma diagnosis, AD, more definite AD (only codes M11z., M11., M111., M114.), AD-related infections, eosinophilic oesophagitis, folliculitis, food allergy, poor sleep, phototherapy, urticaria, and a range of medications used for allergic conditions (adrenaline pens, antibiotics, antihistamines, asthma inhalers, emollients, insomnia drugs, oral corticosteroids, systemic immunosuppressants, topical antibiotics, topical calcineurin inhibitors, and mild, moderate, potent or very potent topical corticosteroids). For the most common codes from each code list, see Table S5 in Supporting Information. From these data, we created different predictor sets. For the main analysis, we used binary variables for each year describing whether an AD diagnosis, diagnosis or treatment, or diagnosis and treatment were present (for other predictor sets, see Table S1).

### Statistical analysis

We calculated summary statistics for the characteristics of the cohort, including AD subtype, sex, social class, and parental AD and asthma, by EHR linkage availability, and calculated the mean and median coverage of the study period in EHRs.

At each timepoint and across all timepoints, we calculated the sensitivity and specificity of the presence of AD symptoms, comparing EHRs to ALSPAC questionnaire responses as the reference standard. We assessed the presence and frequency of AD-related EHRs by ALSPAC AD subtype and assessed intersections (with UpSet plots) of children who had an ALSPAC subtype consistent with having AD (any except unaffected–rare) and children who had an AD diagnosis in EHRs. In secondary analyses, we assessed intersections of children with parent-reported doctor’s AD diagnosis and children who had AD in EHRs. As a comparison, we compared parent-reported doctor’s asthma diagnosis and asthma in EHRs.

Using EHR data up to age 14 years, we classified the AD subtype using multinomial logistic regression methods.^12–14^ We split data into three-quarters training and one-quarter testing data. We normalized numerical data to have an SD of 1 and a mean of 0. We created dummy variables from categorical variables (i.e. converted nominal data into numerical binary model terms). We fitted multinomial logistic regression and tuned the ‘penalty’ and ‘mixture’ hyperparameters (a mixture of 1 specifies a pure lasso model, a mixture of 0 specifies a ridge regression model, and a mixture between 0 and 1 specifies an elastic net model, interpolating lasso and ridge).^15^

We fitted the final model [with the best receiver operating characteristic (ROC) area under the curve (AUC)] to the training set and evaluated the test set. We estimated out-of-sample accuracy and the ROC AUC for each level. We calculated macro-averaged sensitivity and specificity. We plotted a mosaic plot of the confusion matrix to compare the ALSPAC-derived AD subtype (‘truth’) to the AD subtype classified using EHRs (‘prediction’). We then plotted variable importance to visualize which variables were relatively influential in predicting the outcome. We used different sets of variables individually and in combination as predictors in models (Table 1). The study followed the TRIPOD reporting guideline (Appendix S1; see Supporting Information).

**Table 1:** Predictor sets for atopic dermatitis (AD)

	Description	Examples
1	Presence of AD prescription^a^ and diagnostic codes in 1-year windows	Did not have AD diagnostic code between age 0 and 1 year
		Had AD diagnostic code between age 2 and 3 years
		Had AD diagnostic or treatment code between age 5 and 6 years
		Had AD treatment code between age 10 and 11 years
2	Ever/never had code for a given disease or treatment^b^	Had asthma code
		Never had food allergy code
		Had potent topical corticosteroid code
3	How often had codes for a given disease or treatment^b^	Had 2 asthma codes
		Had 0 food allergy codes
		Had 15 potent topical corticosteroid codes
4	Age at first instance of code for a given disease or treatment^b^	Had first asthma code at age 5 years
		Never had a food allergy code
		Had first potent topical corticosteroid code at age 6 years
5	Presence of code for a given disease or treatment^b^ in 1-year windows	Did not have asthma code between age 0 and 1 year
		Had asthma code at age 5 years
		Did not have asthma code at age 6 years
		Had asthma code at age 7 years

^a^Prescriptions for AD include phototherapy, emollients, topical calcineurin inhibitors and mild, moderate, potent or very potent topical corticosteroids. ^b^All disease and treatment codes include allergic rhinitis, asthma-related codes, asthma diagnosis, AD diagnosis, more definite AD diagnosis, AD-related infections, eosinophilic oesophagitis, folliculitis, food allergy, insomnia, phototherapy, urticaria, adrenaline pens, antibiotics, antihistamines, asthma inhalers, emollients, insomnia drugs, oral corticosteroids, systemic immunosuppressants, topical antibiotics, topical calcineurin inhibitors, and mild, moderate, potent or very potent topical corticosteroids.

## Results

### Descriptive statistics and linkage

Of 13 898 individuals in the source data, 11 745 (from both the development and validation cohorts) had been assigned an AD subtype using parent-reported data (including those with the unaffected–rare subtype) and had not withdrawn consent for participation. Of those, 8830 also had linked GP data, of whom 50% were female and 50% were male; these individuals formed the main study cohort. The median EHR coverage of the study period (0–14 years) was 99% (interquartile range 70–99%). In total, 90% of the main study cohort had at least one record from any of the prespecified code lists.

Before the data were split into training and testing sets, there were 356 individuals with the severe–frequent subtype, 716 with moderate–frequent, 1125 with moderate–declining, 872 with mild–intermittent, and 5759 with unaffected–rare.

Those with and without linked primary care data were similar in terms of sex, social class, and parental asthma and AD status (Table 2).^2^

**Table 2:** Characteristics of individuals with and without linked primary care data

Characteristics	EHRs available (*n* = 10 871)	EHRs not available (*n* = 3959)
Atopic dermatitis subtype, *n* (%)		
Severe–frequent	356 (4.0)	117 (4.0)
Moderate–frequent	716 (8.1)	200 (6.8)
Moderate–declining	1125 (13)	327 (11)
Mild–intermittent	872 (9.9)	277 (9.5)
Unaffected–rare	5761 (65)	1994 (68)
Missing	2041	1044
Sex, *n* (%)		
Male	5437 (50)	2126 (54)
Female	5422 (50)	1819 (46)
Missing	12	14
Social class, *n* (%)^a^		
I	1030 (12)	475 (16)
II	3459 (41)	1302 (44)
III(n)	2272 (27)	644 (22)
III(m)	1171 (14)	376 (13)
IV	448 (5.3)	131 (4.5)
V	87 (1.0)	14 (0.5)
Missing	2404	1017
Parental asthma, *n* (%)	1779 (20)	579 (18)
Parental AD, *n* (%)	2705 (30)	885 (28)
	Mean (SD)	Median (IQR)
Start age of EHR data (years)	2.2 (4.6)	0.1 (0.1–2.1)
End age of EHR data (years)	22 (8)	23 (19–30)
EHR coverage (years)^b^	10.9 (5.0)	13.8 (9.8–13.9)
EHR coverage (proportion)^b^	0.78 (0.36)	0.99 (0.70–0.99)

EHR, electronic health record; IQR, interquartile range. Percentages are out of nonmissing records. ^a^Higher social class of either parent. I, professional occupations; II, managerial and technical occupations; III(n), skilled occupations – nonmanual; III(m), skilled occupations – manual; IV, partly skilled occupations; V, unskilled occupations. ^b^Coverage within the study period from 0 to 14 years of age, for those where EHRs were available.

### Agreement between ALSPAC atopic dermatitis parental reports and electronic health records

By age 14 years, the numbers of children who reported AD at least once/twice were 5138 (58% of the cohort)/3383 (38% of the cohort). Of these, 36%/44% also ever had AD, 59%/69% ever had AD or an AD treatment, and 28%/35% ever had AD and an AD treatment in EHRs (Table S6; see Supporting Information).

At timepoints where AD symptoms in ALSPAC were reported, the percentage who also had a record for AD in EHRs up to 1 year before the timepoint ranged from 10% (minimum) at 6 months to 21% (maximum) at 166 months; the percentage who had AD or an AD treatment ranged from 16% at 6 months to 41% at 128 months (Figure S1a; see Supporting Information). When a ‘very bad’ rash was reported, the percentage ranged from 23% at 6 months to 49% at 140 months for AD, and from 35% at 6 months to 86% at 128 months for AD or an AD treatment (Figure S1b).

From secondary analyses, 4222 respondents replied to the question about previous doctors’ AD or asthma diagnoses. Of 2044 with AD in either ALSPAC or EHRs, 676 (33%) had AD in both ALSPAC and EHRs. When a more definite code list for AD was used in EHRs, of 1678 people with AD in either ALSPAC or EHRs, 425 (25%) had AD in both ALSPAC and EHRs. Of 1517 with asthma in either ALSPAC or EHRs, 953 (63%) had asthma in both ALSPAC and EHRs (Figures S2–S4; see Supporting Information).

### Agreement between ALSPAC atopic dermatitis subtypes and electronic health records

**Figure 1:**
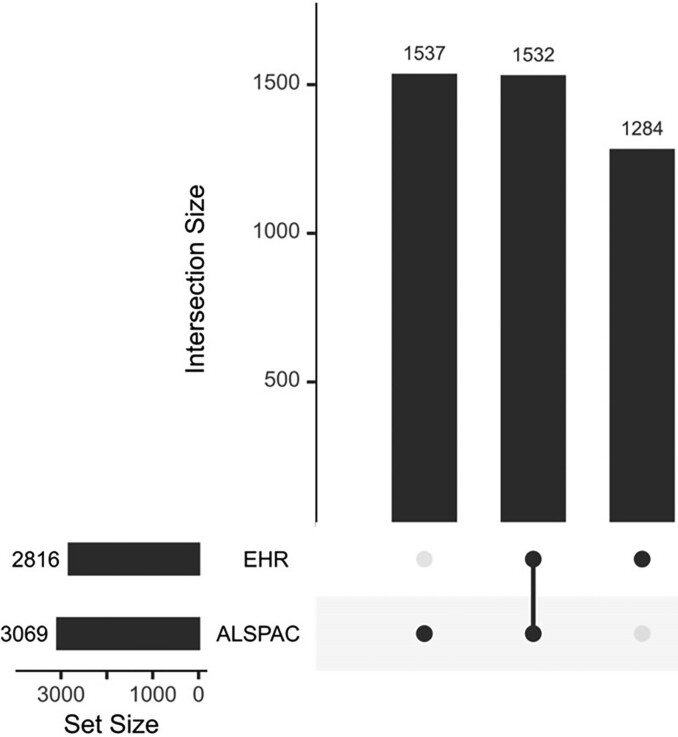
Intersection of individuals with a subtype indicating atopic dermatitis (AD) in ALSPAC and with AD in electronic health records (EHRs). EHR: individuals who have at least one diagnostic code for AD at any time before 166 months (14 years). ALSPAC: individuals who were assigned any of the AD subtypes, except unaffected–rare. Explanation of UpSet plot: of the entire study population (*n* = 8830), 2816 (32%) have AD in EHR, 3069 (35%) have a non-unaffected subtype in ALSPAC, 1284 have AD in EHR only, 1532 have both a non-unaffected subtype in ALSPAC and AD in EHR, and 1537 have a non-unaffected subtype in ALSPAC only.

Of the study cohort (*n* = 8830), 3069 (35%) had a subtype other than unaffected–rare, 2816 (32%) ever had AD in EHRs, and 1532 had both (17% of the study cohort; 35% of the 4353 who had AD in either source) (Figure 1). A sensitivity analysis with a more definite AD code list in EHRs is provided in Figure S5 (see Supporting Information).

**Figure 2:**
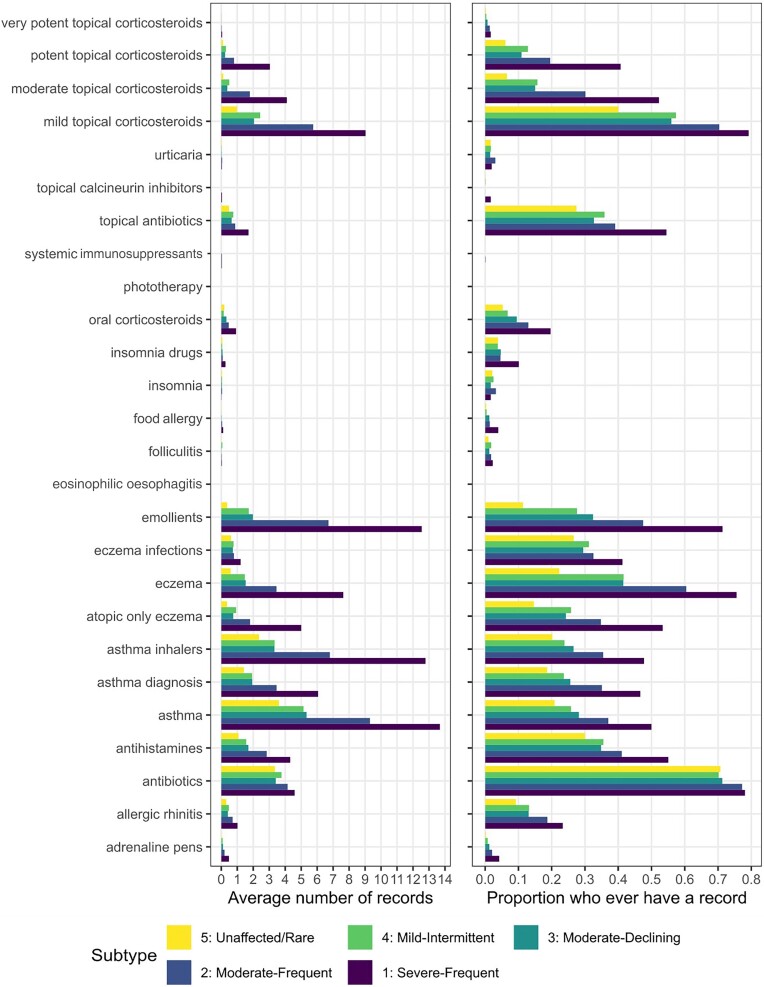
Data in electronic health records by atopic dermatitis subtype. Left: average (mean) number of codes per person. Right: percentage who ever had a record.

The mean number of AD-related records in EHRs and the proportion who had a given record generally increased with more severe and frequent AD subtypes. For example, individuals with the unaffected–rare, mild–intermittent, moderate–declining, moderate–frequent and severe–frequent subtypes had on average (mean) 0.6, 1.5, 1.5, 3.5 and 7.6 records for AD; 22%, 42%, 41%, 60% and 76% ever had a record for AD, respectively. For all variables, see Figure 2. For proportions with AD, AD and AD treatment, and AD or AD treatment, see Table S7 in Supporting Information.

From visual inspection of density plots, there was considerably more overlap between patterns in EHRs by subtype (how often and when records for AD and topical corticosteroids occur) than for symptom and severity reports from ALSPAC by subtype (how often, how severe and when did AD symptoms occur) (Figure S6; see Supporting Information).

### Classifying ALSPAC atopic dermatitis subtypes using electronic health records

**Figure 3:**
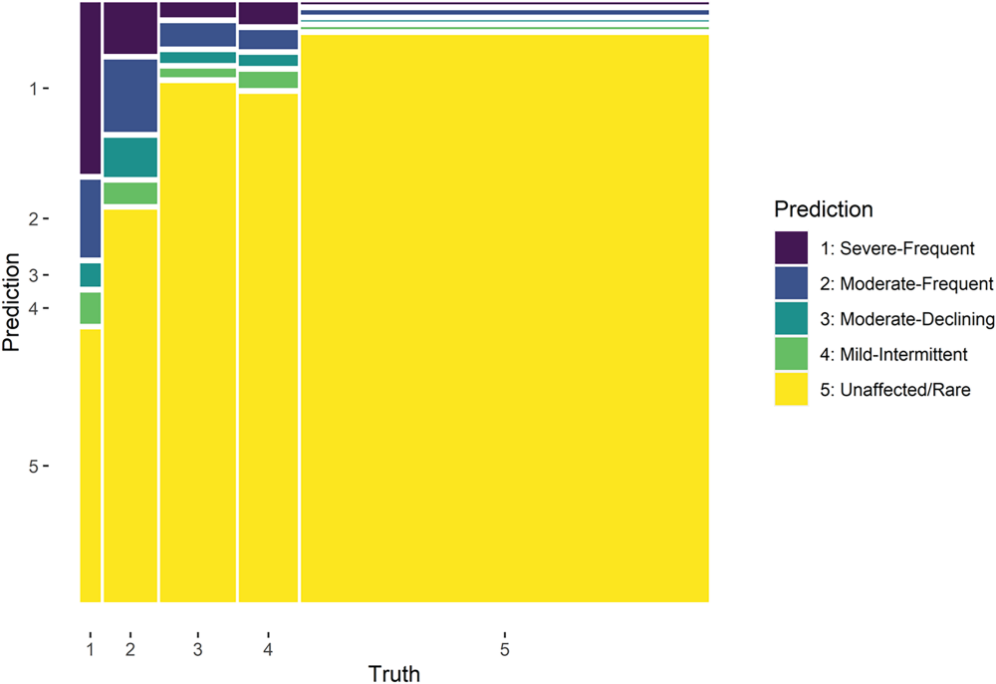
Mosaic plot of the confusion matrix. Mosaic plot showing the predicted (classified) subtypes in rows and coloured, vs. the actual subtypes (truth) in columns. Explanation of plot: almost two-thirds had subtype 5 (unaffected–rare). Almost all of the individuals where the true subtype is 5 (unaffected–rare) were correctly classified as having subtype 5 (unaffected–rare). Only a small proportion of individuals with subtypes 3 (moderate–declining) and 4 (mild–intermittent) were correctly classified, with most being classified as subtype 5 (unaffected–rare). Almost one-third of individuals with subtype 1 (severe–frequent) were correctly classified; however, almost half were classified as having subtype 5 (unaffected–rare).

There were 6622 observations used in the final model with predictor set 1 (AD diagnosis and/or treatment codes for each year). The tuned model hyperparameters were 0 for mixture (i.e. ridge regression) and 1 × 10^−10^ for penalty. Fitting the tuned model to the training data and evaluating the testing data showed an ROC AUC of 0.65 and model accuracy of 0.68. The sensitivity was 0.29 and the specificity was 0.83 (both macro-averaged). The individual ROC was best for the severe–frequent subtype, and worst for the moderate–declining and mild–intermittent subtypes (Figure S7; see Supporting Information). The model classified more people as having the unaffected–rare subtype than actually had the unaffected–rare subtype (Figure 3; and Table S8; see Supporting Information).

Predictive performance was similar with different predictor sets, and in sensitivity analyses (Table 3). The sensitivity analyses excluded observations (i) from individuals who did not have GP data available before age 2 years and up to at least age 13 years; (ii) where responses were not recorded for all ALSPAC questionnaires; and (iii) from individuals with the unaffected–rare subtype. The performance when outcome variables with fewer subtype categories were used was somewhat improved (e.g. ROC AUC of 0.72 when moderate–frequent, moderate–declining and mild–intermittent were combined) (Table S9; see Supporting Information).

**Table 3:** Metrics by predictor set used

Predictor set	ROC AUC^a^	Accuracy	Sensitivity^b^	Specificity^b^
1: Presence of AD prescription and diagnostic codes in 1-year windows	0.65	0.68	0.29	0.83
2: Ever/never had code for a given disease or treatment	0.63	0.66	0.28	0.82
3: How often had code for a given disease or treatment	0.63	0.66	0.25	0.82
4: Age of first occurrence for a given disease or treatment	0.64	0.66	0.27	0.83
5: Presence of code for a given disease or treatment in 1-year windows	0.63	0.65	0.27	0.83
1 + 3	0.68	0.67	0.31	0.83
1 + 3 + 5	0.64	0.67	0.30	0.83

^a^Receiver operating characteristic area under the curve. This was averaged using the method of Hand and Till.^30^  ^b^Sensitivity and specificity are macro-averaged.

When using ever/never or count variables to classify AD subtypes, both resulted in more parsimonious lasso regression models with the best ROCs. The most important factors in predicting the outcome were the number of potent topical corticosteroids (for count variables), and ever having records for emollients, moderate topical corticosteroids, or eczema (for ever/never variables) (Figure S8; see Supporting Information).

## Discussion

Our main findings were, first, that individuals were more likely to have AD recorded in EHRs if their parents had reported more frequent or more severe AD symptoms in ALSPAC. Secondly, while those with more severe subtypes had greater prevalence and more records for AD-related variables in EHRs, there was considerable overlap between patterns in EHRs by subtype, and there was disagreement between having a subtype consistent with having AD, and having AD in EHRs. Thirdly, this disagreement and overlap explain why using data from EHRs to classify ALSPAC-derived subtypes results in poor sensitivity predictions.

Both ALSPAC and EHRs may have wrongly classified AD and AD severity trajectories. Parental reports in ALSPAC may have been subject to measurement error and subject to differential parental perception of disease severity, which may have resulted in individuals’ assigned ALSPAC subtype not corresponding to their actual severity trajectory. EHRs may be subject to several pitfalls concerning variable definitions,^16^ and may not have adequately captured AD. For example, EHRs could miss AD diagnoses; in people with less severe AD not consulting the GP, where GPs do not re-record diagnoses that had already been recorded previously, or where diagnoses from specialist care are not recorded in primary care.

EHRs could also misclassify individuals who do not have AD as having AD. An example is where GPs use diagnostic codes for AD to record other non-AD rashes; however, there was still considerable disagreement when AD in EHRs was defined using a more definite code list. EHRs may also miss over-the-counter AD therapies, and only contain information on whether a therapy was prescribed, not on whether and how therapies were used.

With prevalence estimates of AD ranging from 10% to 30% in other studies,^17–20^ having one of the subtypes consistent with having AD (35%), and ever having a record for AD in EHRs (32%) may capture slightly more individuals than actually have AD. However, we can also not conclude that the 17% who had AD in both sources represent a cohort who truly have AD, as this cohort would exclude some individuals where EHRs clearly indicate AD or whose parents frequently reported very bad AD symptoms in ALSPAC.

Other results were more consistent with clinical expectations. For example, as not every child with AD symptoms should be considered as having AD, only about one-third of individuals who report AD symptoms once in ALSPAC had an AD diagnostic code in EHRs. The proportion increased for those where rashes were reported at least twice. At timepoints where a ‘very bad’ rash was reported, up to 86% at 128 months had records for an AD diagnosis or treatment in EHRs, suggesting that most did receive care. However, there was variation by age, with the smallest proportion of EHR diagnoses and treatments in those who reported rashes at the earliest timepoints, even though this may be the period of highest actual prevalence.^17,18,20^ The variation is possibly due to different approaches to prescribing, or different diagnostic codes used for infants than for older children.

From secondary analyses, agreement (diagnosis in both sources) between parent-reported doctors’ diagnoses from ALSPAC and records in EHRs was much better, albeit not perfect, for asthma (63%) compared with AD (33%). This suggests that the disagreement found in this study may be a problem particular to AD. Reasons for better agreement for asthma compared with AD may include more guideline-driven care with more regular follow-up for asthma, inclusion in the Quality and Outcomes Framework,^21^ the potentially life-threatening nature of asthma symptoms, and different social perceptions. Reasons for disagreement, which may also explain the remaining disagreement for asthma, may include parents not recalling that their child had been diagnosed, or the questionnaire leading parents to only report more recent diagnoses. The question of whether ‘a doctor has ever actually said [the child] has eczema’ followed up a form recording illnesses in the past 12 months.

We did not find any other studies where classifying disease subtypes in ALSPAC was attempted using EHRs. Primary care EHRs linked to ALSPAC have previously been used to assess and predict ALSPAC-derived common mental health disorder diagnoses, where EHRs generally underestimated the prevalence of mental health conditions compared with ALSPAC.^22^ Another study of ALSPAC and linked primary care data investigated the performance of parent-reported responses in identifying physician-confirmed asthma in EHRs. That study showed high agreement (88.5% sensitivity and 95.7% specificity).^23^

Previous studies have used latent class analysis to identify childhood AD subtypes in birth cohort studies,^24,25^ including ALSPAC,^26^ without incorporating reports of symptom severity. Future research may evaluate whether subtypes based on a trajectory of symptom presence, but not severity, might be better replicated in EHR data.

Poor agreement concerning AD may also be an issue in other cohort studies that are linked to EHRs. For example, a recent study analysed UK Biobank participants (aged 40–69 years) who either self-reported previous AD or had records for AD in their linked primary care EHR data. Of these, only a minority had AD in both data sources.^3^

Not all individuals had EHR coverage for the whole study period, as some AD-related codes might have been missed in EHRs. However, predictive performance was not improved when individuals with less complete EHR coverage were excluded. Access to EHRs was also restricted to events with a code in one of the prespecified code lists, and we might have missed codes that could have helped predictive performance.

While multinomial logistic regression has been used for multiclass classification in previous studies with similar aims,^22^ utilizing other machine learning methods might have yielded better predictive performance. While lasso regression allows shrinking of coefficients to 0 (i.e. dropping nonpredictive variables from the model), ridge or elastic net have advantages if covariates are highly correlated.^27^ This was probably the case in our setting, which is why we tuned the mixture parameter.

Future research should assess agreement between ALSPAC and linked EHRs for other conditions, as was already done in this study for asthma, and in other studies for conditions such as anxiety and depression.^22^ Agreement concerning AD should also be examined across other data sources, as our findings may not be generalizable to other settings.^3^ Being able to compare agreement across conditions and data sources may help establish whether the disagreement concerning disease status found in this study is specific to AD, or specific to a given data source such as ALSPAC. Future studies that use ALSPAC and/or linked EHRs to define AD should acknowledge uncertainty surrounding AD subtypes and AD diagnoses, and the implications of potential misclassification on study findings. Future prospective cohort studies may more reliably capture AD status by implementing AD measures that have been previously validated, such as those recommended in the Harmonising Outcome Measures for Eczema statement.^28^ Finally, future EHR studies should clearly report AD definitions and ideally make use of validated definitions.^29^

## Conclusions

While AD subtypes correlated with several AD-related variables in EHRs, there was considerable overlap in EHRs on an individual level, and even disagreement between data sources on whether children had AD or not, precluding sensitive classification using EHRs. While using multiple data sources may help more accurately determine who has AD, we cannot conclude from this study alone that the intersection of AD in ALSPAC and EHRs represents true cases of AD. Further research validating AD-related study information is needed. When interpreting research on AD in either ALSPAC or UK primary care EHRs, it needs to be kept in mind that people considered as having AD in one source may not be considered as having AD in another.

## Supplementary Material

llae196_Supplementary_Data

## Data Availability

All analytical code and code lists used for this study are available at https://zenodo.org/records/12731490. Access to ALSPAC data is through a system of managed open access. Information about access to these data is given on the study website (http://www.bristol.ac.uk/alspac/researchers/access) and in the data management plan (https://www.bristol.ac.uk/media-library/sites/alspac/documents/researchers/data-access/alspac-data-management-plan.pdf). The data used for this submission will be made available on request to the executive (alspacexec@bristol.ac.uk). The datasets presented in this article are linked to ALSPAC project number B2510; please quote this project number during your application. For additional details on accessing the ALSPAC linkage data, see http://www.bristol.ac.uk/alspac/researchers/our-data/linkage.
