# Supplementary material for: Disagreement concerning atopic dermatitis subtypes between an English prospective cohort (ALSPAC) and linked electronic health records
Source: Clin Exp Dermatol. 2024 May 16;49(12):1537–46. doi: 10.1093/ced/llae196 (PMC11583923; doi:10.1093/ced/llae196)
Supplement: llae196_Supplementary_Data [file llae196_supplementary_data.docx]

# Appendix

## eFigure 1: Sensitivity at each timepoint


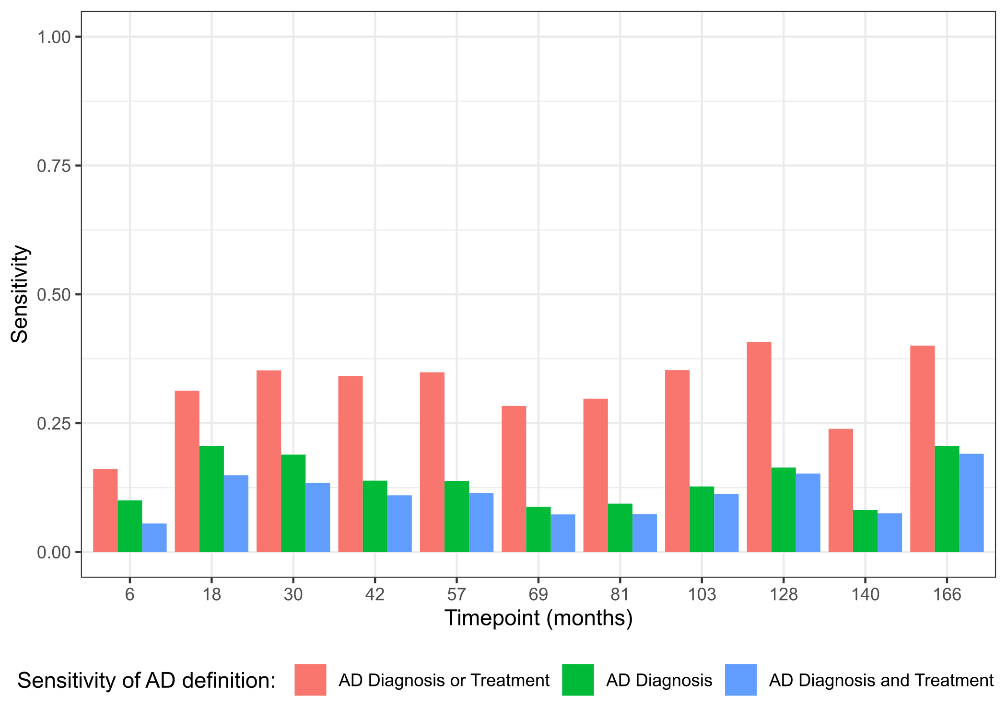
(a)
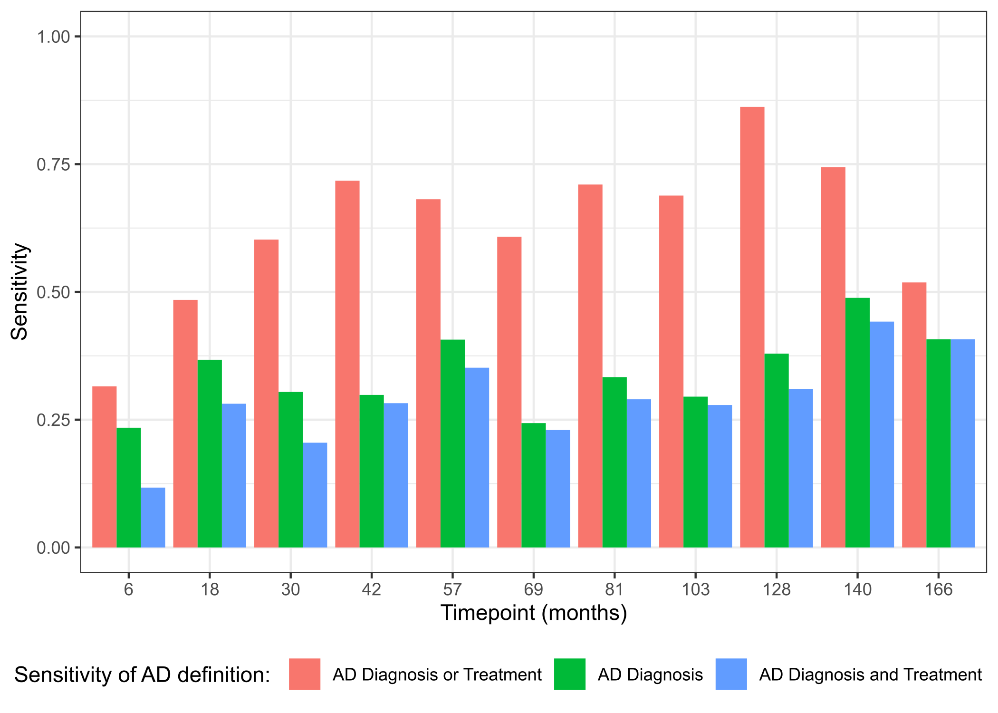
 (b)

*Figure Legend: Sensitivity at each timepoint, comparing (a) a positive ALSPAC symptom report, (b) a positive ALSPAC symptom report where a “very bad” rash was reported, as the reference standard to information from EHRs from the past 12 months. E.g. (a) at 30 months, of those who reported flexural dermatitis in ALSPAC, x% had an AD diagnosis, x% had an AD diagnosis or treatment, and x% had an AD diagnosis and treatment in the past year. Specificity, i.e., the proportion of those who didn’t report AD symptoms in ALSPAC and also didn’t have an AD diagnosis in EHRs was >85% across all timepoints and definitions of AD in EHRs.
Dx_and_rx: AD diagnosis and prescription, Dx_or_rx: AD diagnosis or prescription, dx: AD diagnosis*

## eFigure 2: UpSet plot showing the intersection of parent-reported doctor’s AD diagnosis in ALSPAC and AD in EHRs


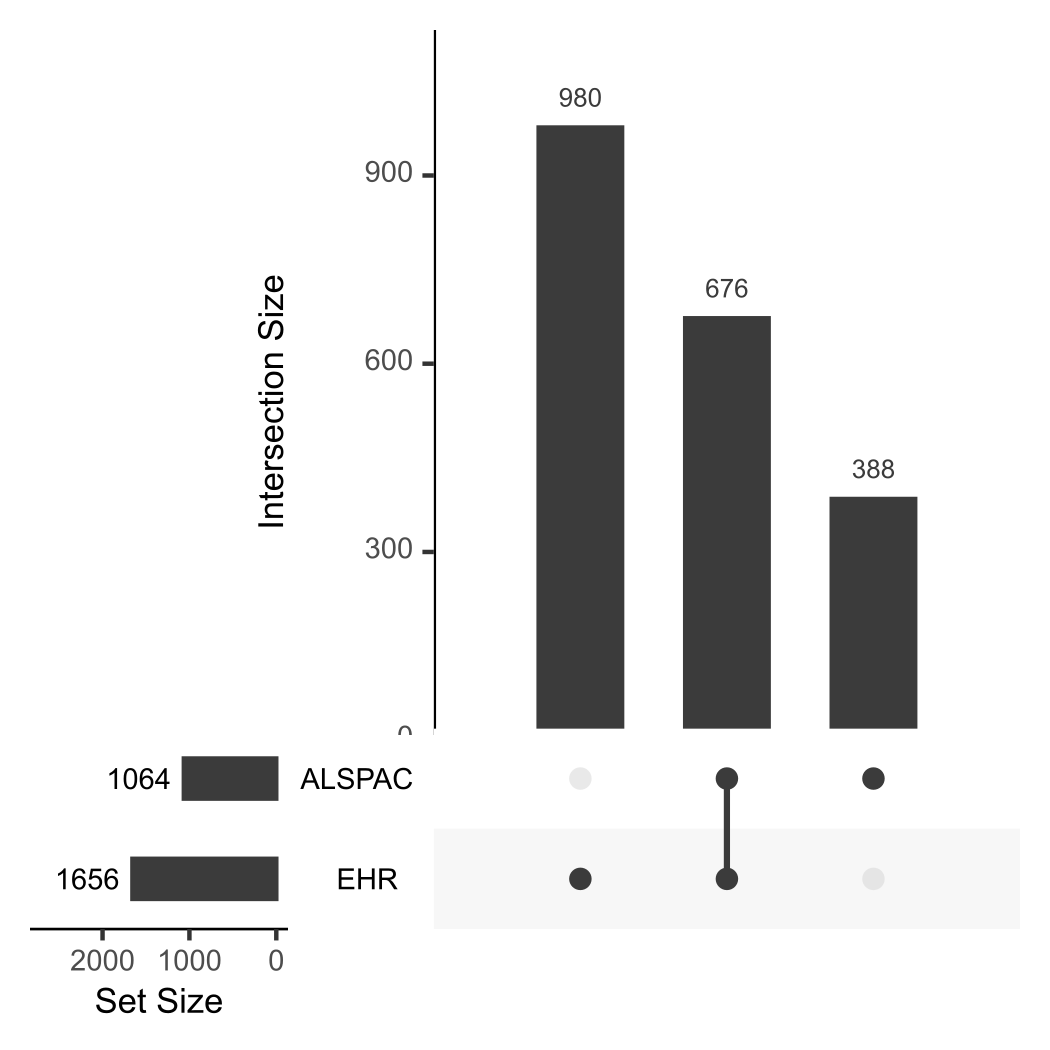


*Figure Legend: ALSPAC=Individuals, whose parents or carer responded “Yes, eczema” or “Yes, asthma and eczema” to the question if the child had ever been diagnosed by a doctor with asthma or eczema at 166 months (exact wording of question: “Has a doctor ever actually said that he/she has asthma or eczema?); EHR=Individuals that have at least one record for AD at any time before 166 months (14 years). Both ALSPAC and EHR from are from a total of 4,222 that responded to the question in ALSPAC.*

## eFigure 3: UpSet plot showing the intersection of parent-reported doctor’s AD diagnosis and AD, using a more definite AD codelist, in EHRs


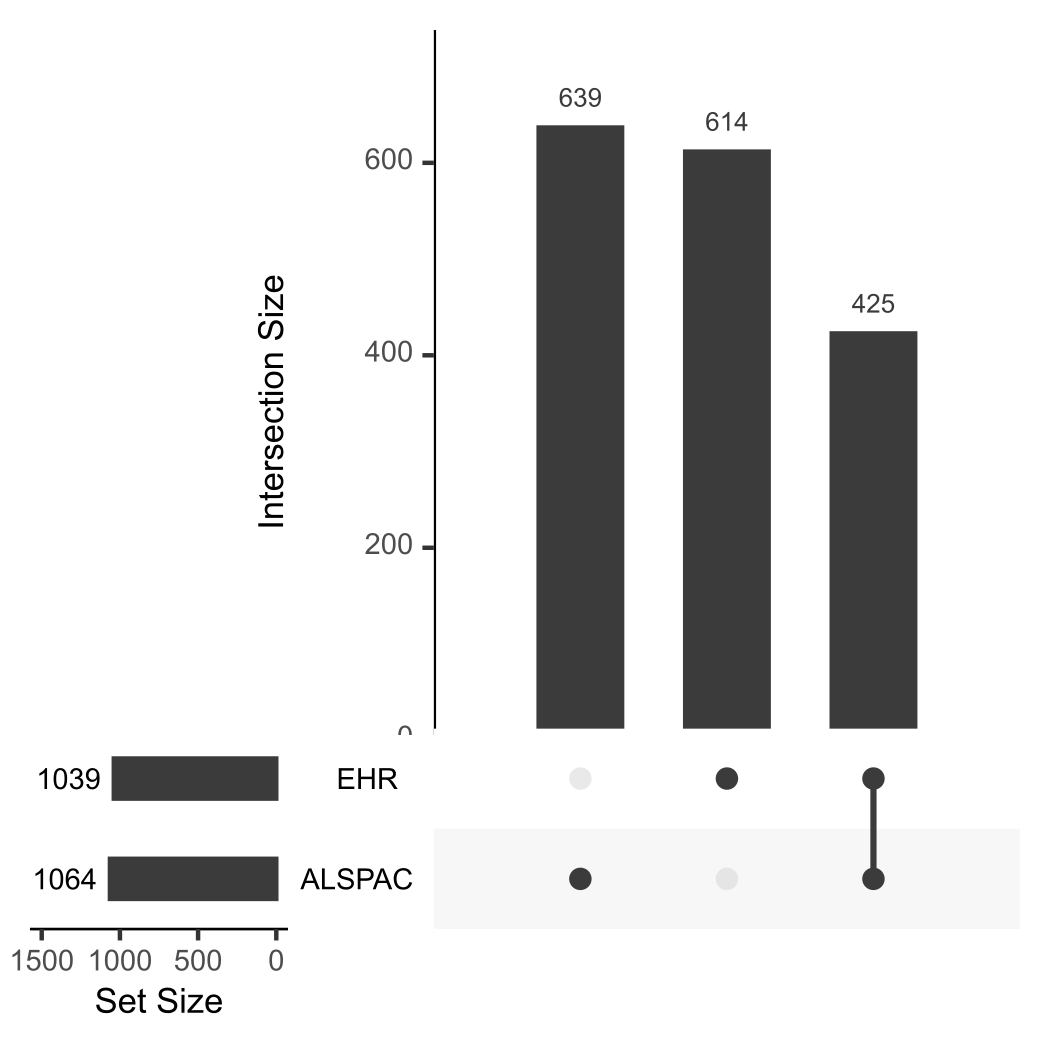


*Figure Legend: ALSPAC=Individuals, whose parents or carer responded “Yes, eczema” or “Yes, asthma and eczema” to the question if the child had ever been diagnosed by a doctor with asthma or eczema at 166 months (exact wording of question: “Has a doctor ever actually said that he/she has asthma or eczema?); EHR=Individuals that have at least one record for “M11z. atopic dermatitis/eczema”, “M11.. atopic dermatitis and related”, “M111. atopic dermatitis nos” or “M114. Allergic (intrinsic) eczema” at any time before 166 months (14 years). Both ALSPAC and EHR from are from a total of 4,222 that responded to the question in ALSPAC.*

## eFigure 4: UpSet plot showing the intersection of parent-reported doctor’s asthma diagnosis in ALSPAC and asthma in EHRs


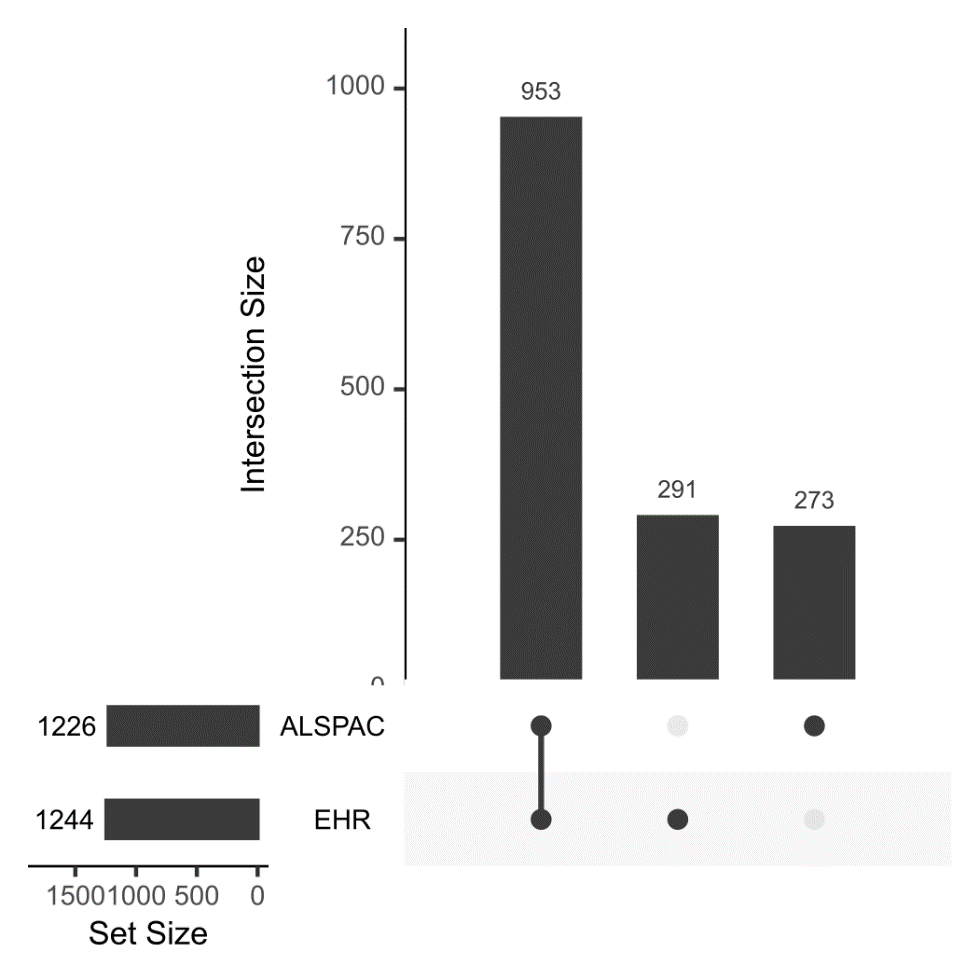


*Figure Legend: ALSPAC=Individuals, whose parents or carer responded “Yes, asthma” or “Yes, asthma and eczema” to the question if the child had ever been diagnosed by a doctor with asthma or eczema at 166 months (exact wording of question: “Has a doctor ever actually said that he/she has asthma or eczema?); EHR=Individuals that have at least one record for asthma at any time before 166 months (14 years). Both ALSPAC and EHR from are from a total of 4,222 that responded to the question in ALSPAC.*

## eFigure 5: UpSet plot showing the intersection having a subtype indicating AD in ALSPAC and having AD in EHRs, using a more definite AD codelist


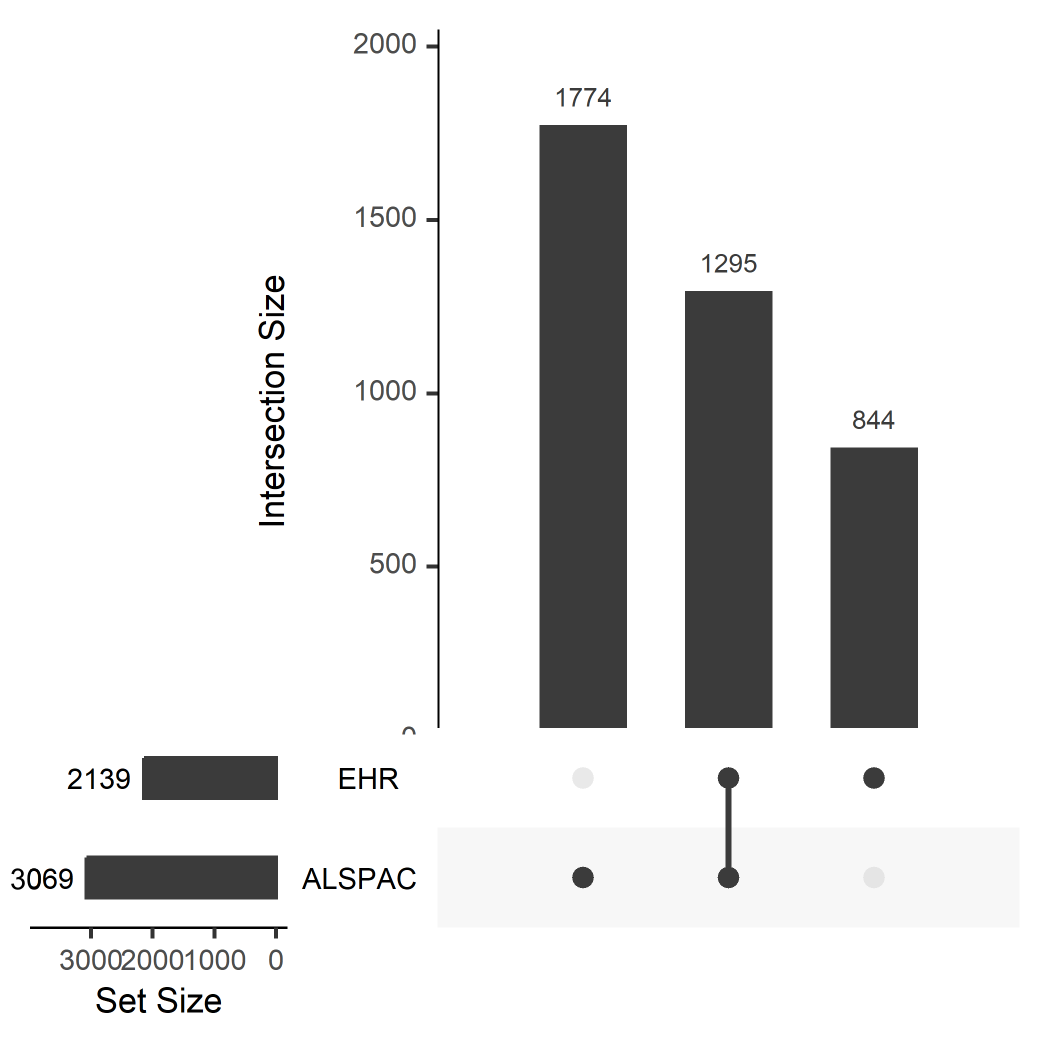


*Figure Legend: EHR=Individuals that have at least one record for “M11z. atopic dermatitis/eczema”, “M11.. atopic dermatitis and related”, “M111. atopic dermatitis nos” or “M114. Allergic (intrinsic) eczema” at any time before 166 months (14 years); ALSPAC=Individuals, whose parents or carer responded “Yes, eczema” or “Yes, asthma and eczema” to the question if the child had ever been diagnosed by a doctor with asthma or eczema at 166 months (exact wording of question: “Has a doctor ever actually said that he/she has asthma or eczema?).*

## eFigure 6: Density plots


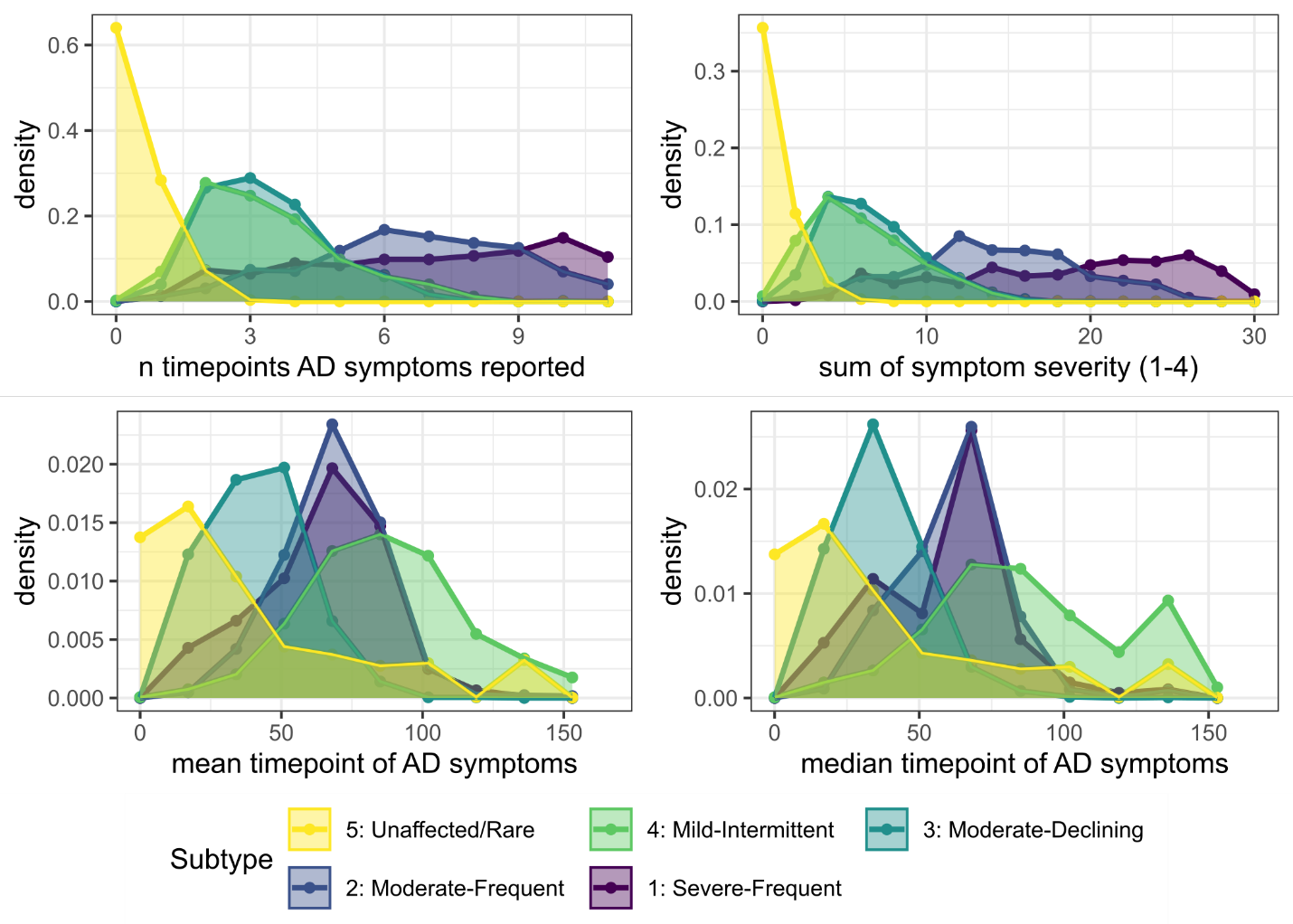

(a) from ALSPAC symptom and severity reports


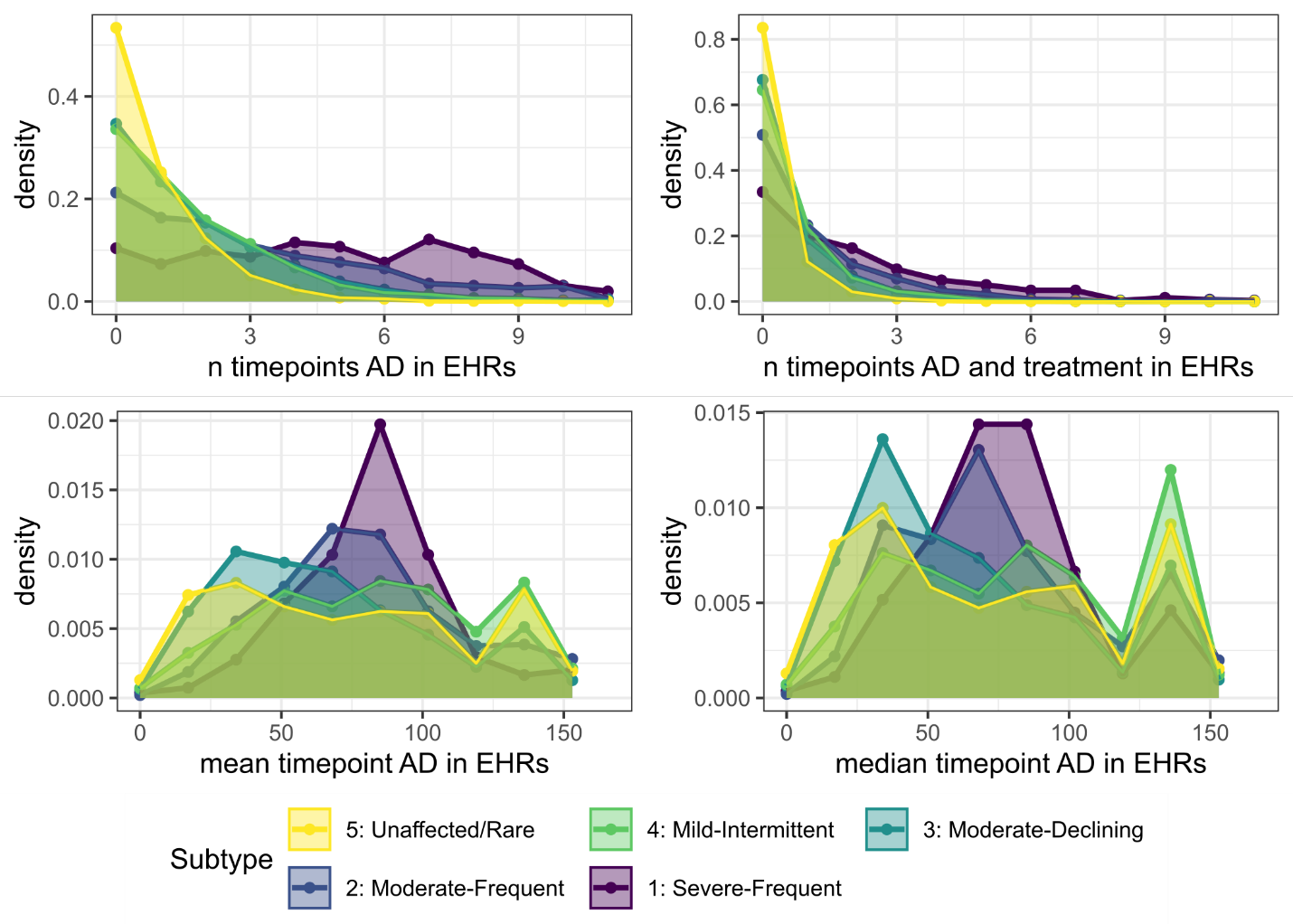

(b) from time-point specific EHR variables


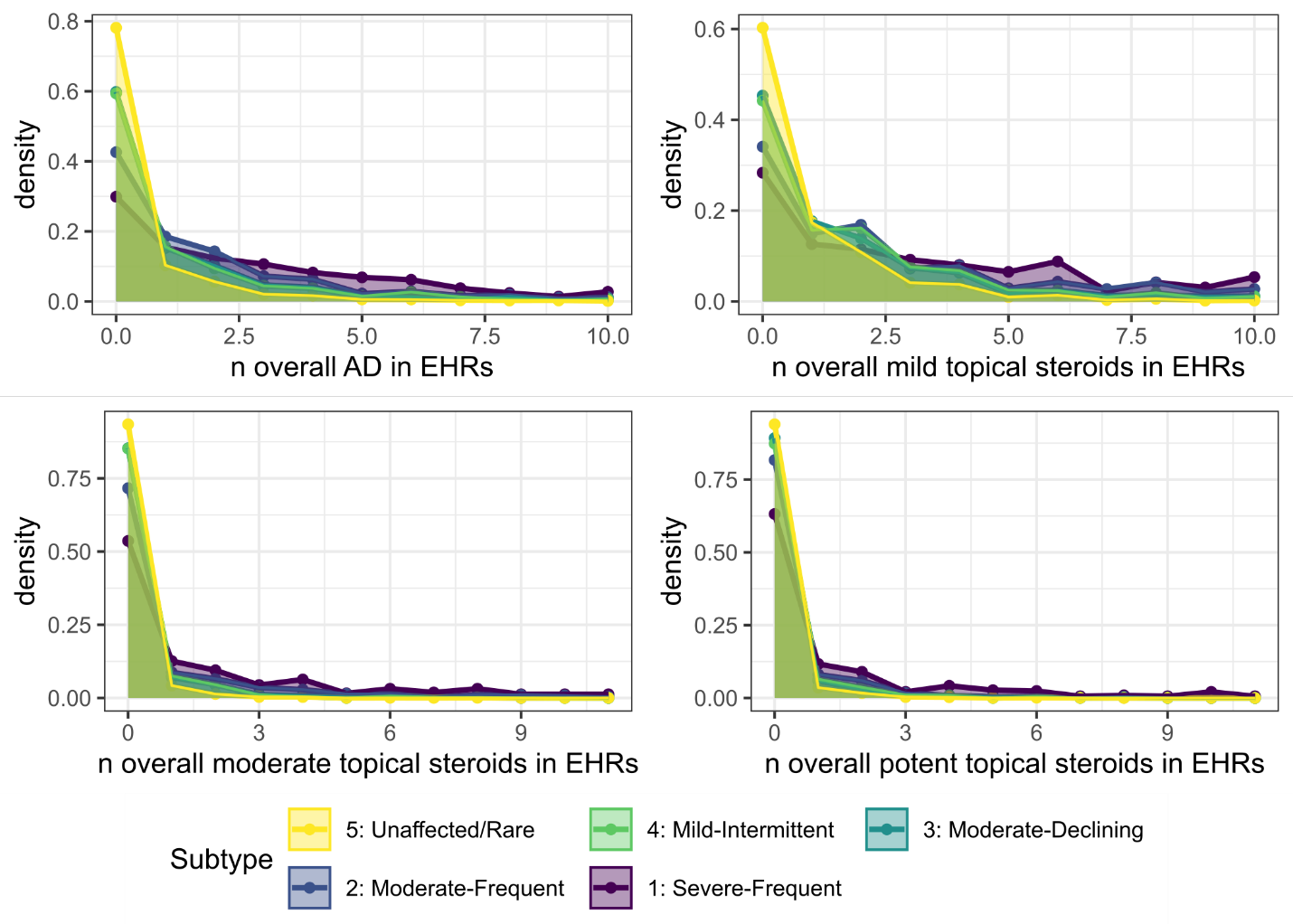

(c) from overall EHR variables

*Figure Legend: Density plots showing how many individuals had a certain number/sum/mean/median for variables from: (a) the original ALSPAC AD symptom and severity reports; (b) timepoint-specific variables in EHRs; (c) overall count variables in EHRs (for (c), x-axis limit set at 10, however individuals could have more than 10 records).
Mean and median timepoints in (a) and (b) are in months. Density describes the distribution of values by subtype (e.g., in (a), of those with the Unaffected/Rare subtype, about 60% had AD at 0 timepoints, about 30% had AD at 1 timepoint, and about 10% had AD at 2 timepoints).*

## eFigure 7: Receiver operating characteristic (ROC) curves


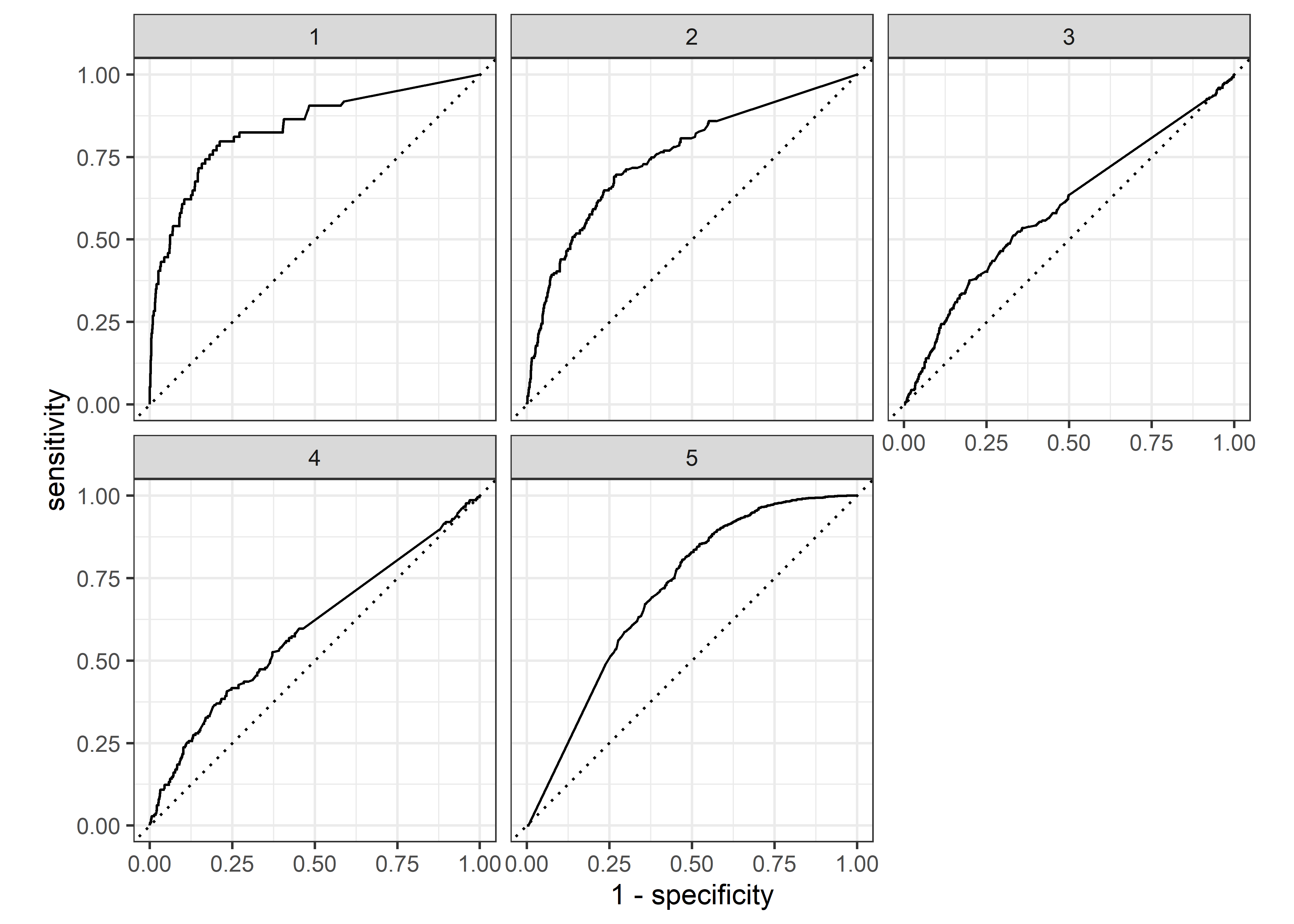


*Figure Legend: Receiver operating characteristic (ROC) curves, illustrating the diagnostic ability of a binary classifier system as its discrimination threshold is varied.*

## eFigure 8: Variable Importance Plot


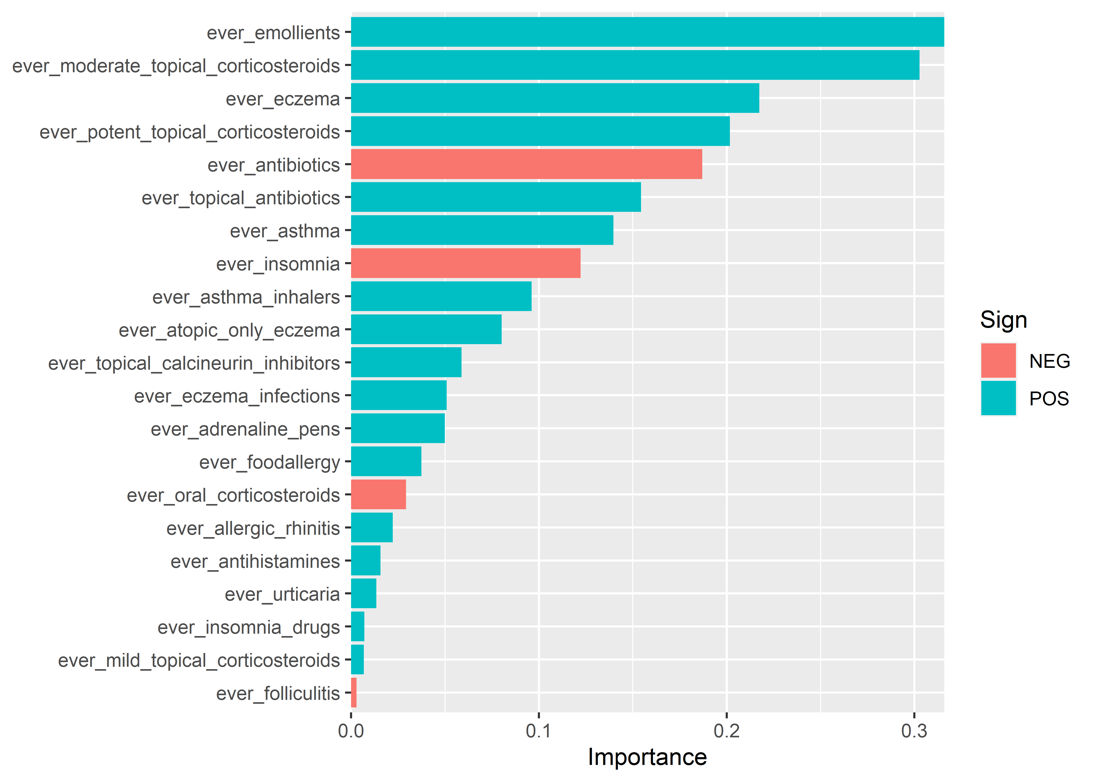


(a: when using count variables to classify AD subtype)


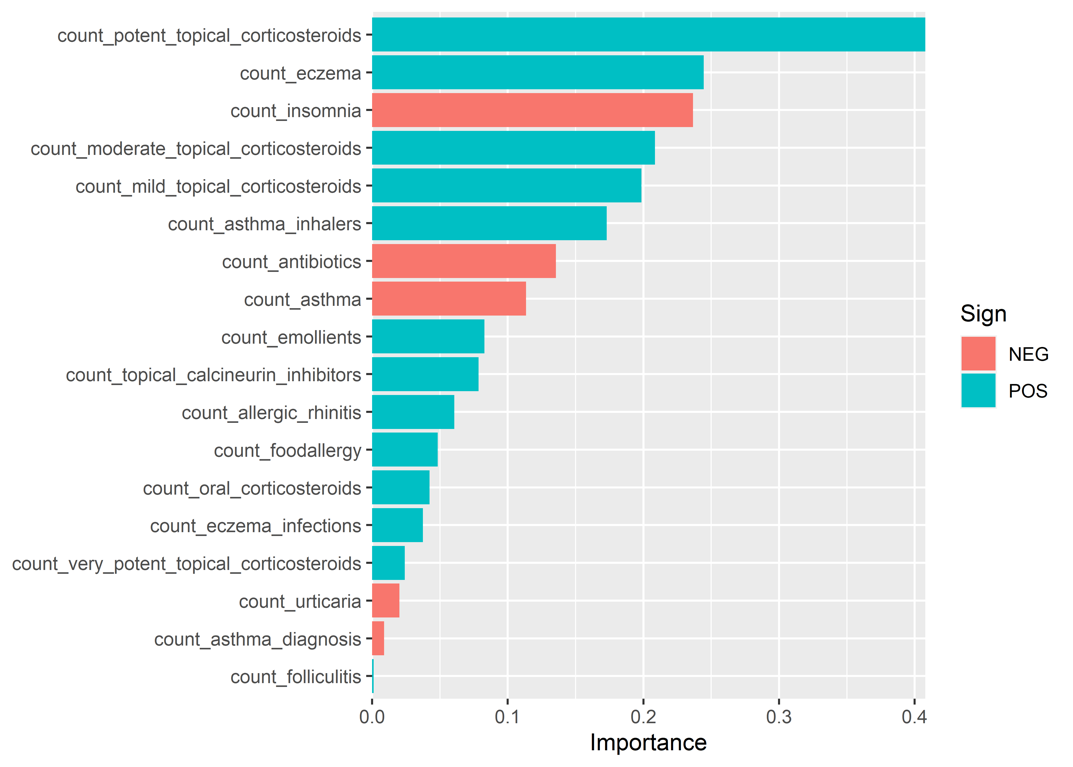


(b: when using ever/never variables to classify AD subtype)

Figure Legend: Variable Importance plots showing the relative importance of a variable in predicting the outcome.

## eTable 1: Codelists

| Variable | Description |
| --- | --- |
| allergic rhinitis | allergic rhinitis and related allergies, including hay fever, dust (mite), pollen and animal allergies |
| asthma | codes that only people with current asthma would have recorded (including things like severity assessments, clinic visits, management plans, etc..), excluding codes where it is equally likely that the person does not have asthma (e.g. asthma screening) |
| asthma diagnosis | asthma diagnosis codes, excluding asthma related codes like clinic visits, assessments, etc... |
| atopic dermatitis | atopic dermatitis/atopic eczema, excluding codes for unspecific forms of eczema |
| atopic dermatitis related infections | skin infections related to atopic dermatitis, excluding secondary complications of these infections (e.g. ocular, systemic infections, etc...) |
| eosinophilic eosophagitis | Eosinophilic eosophagitis |
| folliculitis | codes for infectious folliculitis, not inflammatory diseases (e.g. folliculitis deplians, decalvans, etc...) |
| food allergy | Food allergies, not including intolerances (e.g. lactose intolerance) |
| insomnia | Insomnia related codes |
| phototherapy | Phototherapy, including photochemotherapy, excluding photodynamic therapy (usually a therapy for skin cancers) |
| urticaria | atopy related urticaria, excluding drug induced urticaria or mast cell disorders (like urticaria pigmentosa, neonatorum, etc...) |
| adrenaline pens | Adrenaline auto-injectors ("EpiPens") |
| antibiotics | oral antibiotics used to treat skin infections, excluding topical |
| antihistamines | non-specific list of antihistamines, including those prescribed for sleep or coughs and colds (e.g. those with paracetamol, ibuprofen), excluding drugs for nausea, vomiting and vertigo which can be part of the same substance class |
| asthma inhalers | Asthma inhalers |
| emollients | Emollients and moisturisers, including all from https://bnf.nice.org.uk/drugs/emollient-creams-and-ointments-paraffin-containing/medicinal-forms/ |
| insomnia drugs | Medicines for insomnia including hypnotics, benzodiazepines, anxiolytics, herbal remedies, and sedating antihistamines |
| mild topical corticosteroids | Mild topical corticosteroids as per https://bnf.nice.org.uk/treatment-summaries/topical-corticosteroids/ |
| moderate topical corticosteroids | Moderate topical corticosteroids as per https://bnf.nice.org.uk/treatment-summaries/topical-corticosteroids/ |
| oral corticosteroids | Oral corticosteroids and glucocorticoids, excluding oestrogen steroid hormones (e.g. oestradiol, estrone, exemestane) and steroids with predominantly mineralocorticoid activity (e.g. fludrocortisone) |
| potent topical corticosteroids | Potent topical corticosteroids as per https://bnf.nice.org.uk/treatment-summaries/topical-corticosteroids/ |
| systemic immunosuppressants | Systemic immunosuppressants, including cyclosporine, azathioprine, methotrexate, mycophenolate, tacrolimus |
| topical antibiotics | Topical antibiotics for impetigo and other skin infections, including Fusidin and Mupirocin according to NICE Guidance (https://www.nice.org.uk/guidance/ng153/chapter/recommendations#choice-of-antimicrobial) |
| topical calcineurin inhibitors | Topical calcineurin inhibitors |
| very potent topical corticosteroids | Very potent topical corticosteroids as per https://bnf.nice.org.uk/treatment-summaries/topical-corticosteroids/ |

## eTable 2: Termsets

| Searchterms | Exclusionterms |
| --- | --- |
| allergic rhinitis | |
| "allergic rhinitis", "allergic rhinosinusitis", "pollinosis", "perennial rhinitis", "hay fever", "cat allergy", "dander allergy", "house dust allergy", "dog allergy", "feather allergy", "animal allergy" | "fh:", "h/o:", "family history", "eye drops", "past history", "preps" |
| asthma diagnosis | |
| "\"asthma\"", "asthma", "status asthmaticus" | "monitoring", "number of", "limits walking", "treatment compliance", "daytime symptoms", "night symptoms", "attendance", "currently", "restricts", "admission", "medication", "severity", "management", "limiting", "limits", "disturbing", "disturbs", "causing", "overlap", "administration", "monitored", "monitor", "review", "trigger", "control", "prophylaxis", "nedocromil", "sodium", "causes", "education", "drug", "reporting", "indicators", "clinic", "family history", "adverse reaction", "specialist", "leaflet", "screening", "resolved", "study", "detergent", "assessment", "action plan", "fh:", "h/o:", "suspected", "society", "absent", "follow-up", "symptoms", "at risk of", "questionnaire" |
| atopic dermatitis related infections | |
| "molluscum contagiosum", "herpes simplex", "hsv", "eczema herpe*", "impetig*", "varicelliform eruption", "molusc*", "cold sore", "mollusc*", "cellulitis", "staph* skin", "whitlow", "scrum pox", "herpesviral vesicular dermatitis", "herpetic gingivostomatitis", "herpetic stomatitis", "herpes labialis" | "neonatorum", "ophthalmic", "meningitis", "keratitis", "iridocyclitis", "pneumonia", "otitis", "septicaemia", "genital", "detection", "therapy", "pharynx", "vocal cords", "larynx", "seminal vesicle", "eosinophilic cellulitis", "periurethral", "gonococcal", "serologic test", "cream", "encephalitis", "polymerase chain reaction", "level", "virus isolation", "parametritis", "pelvic cellulitis", "oral cellulitis", "impetigo herpetiformis", "floor of mouth", "soft tissue cellulitis", "visceral herpes", "antigen" |
| eosinophilic eosophagitis | |
| eosinophilic oesophagitis | NONE |
| folliculitis | |
| folliculitis | "sycosis", "depilans", "abscedens et suffodiens", "ulerythematosa", "decalvans" |
| foodallergy | |
| "food allergy", "egg allergy", "fruit allergy", "tomato allergy", "banana allergy", "soya allergy", "strawberries allergy", "strawberry allergy", "mushroom allergy", "shellfish allergy", "seafood allergy", "fish allergy", "wheat allergy", "nut allergy", "peanut allergy" | none |
| insomnia | |
| "insomnia", "poor sleep", "delayed sleep", "restless sleep", "sleep disorder*", "sleep disturb*", "sleep dysfunction*", "sleep problem*" | "nonorganic", "sleep apnoea", "non-organic", "arousal", "emotional", "asthma", "eating", "chronic obstructive pulmonary disease" |
| phototherapy | |
| "puva", "light therapy", "phototherapy", "ultraviolet b therapy", "ultraviolet a therapy" | "device", "complication" |
| urticaria | |
| "urticaria", "hives", "nettle rash" | "test", "amyloid nephropathy with deafness and urticaria", "factitial", "drug induced", "menstrual", "familial febrile", "pigmentosa", "solar", "neonatorum", "due to serum" |
| antibiotics | |
| "flucloxacillin", "clarithromycin", "erythromycin", "amoxicillin" | "cutaneous", "ointment", "solution", "overdose", "adverse reaction", "test", "resistant", "immunoglobulin", "sensitivity", "allergy", "measurement", "poisoning", "parenteral", "lotion", "ophthalmic" |
| antihistamines | |
| "antihistamine", "antazoline", "carbinoxamine", "diphenhydramine", "pyrrobutamine", "tripelennamine", "brompheniramine", "mepyramine", "methapyrilene", "triprolidine", "dexchlorpheniramine", "hydroxyzine", "clemastine", "chlorphenamine", "fexofenadine", "levocabastine", "ketotifen", "chlorpheniramine", "phenyltoloxamine", "meclozine", "pheniramine", "loratadine", "dexbrompheniramine", "dimetindene", "bromazine", "diphenylpyraline", "piprinhydrinate", "homochlorcyclizine", "clocinizine", "bromodiphenhydramine" | "adverse reaction", "allergy", "measurement", "trimethobenzamide", "cinnarizine", "flunarizine", "poisoning", "prophylaxis", "overdose" |
| emollient | |
| "emollient", "animal fat substance", "petrolatum substance", "wool fat", "water in oil agent substance", "spermaceti", "titanium dioxide substance", "waxes substance", "yellow wax", "white wax substance", "cocoa butter substance", "cold cream substance", "white lotion substance", "colophony substance", "primin substance", "styrax substance", "tar substance", "alene substance", "methylated naphthalene substance", "wood preservative substance", "balsam substance", "cetylpyridinium substance", "prophyllin" | "adverse", "procedure", "cathartic", "disorder", "blood group", "margarine", "control" |
| insomnia drugs | |
| "zolpidem", "stilnoct", "zopiclone", "zimovane", "chloral hydrate", "cloral betaine", "loprazolam", "lormetazepam", "flurazepam", "dalmane", "nitrazepam", "mogadon", "temazepam", "diazepam", "diazemuls", "stesolid", "lorazepam", "ativan", "oxazepam", "promethazine hydrochloride", "phenergan", "sominex", "melatonin", "syncrodin", "slenyto", "circadin", "hydroxyzine", "ramelteon", "tasimelteon" | "overdose", "poisoning", "adverse reaction", "level", "dependence", "allergy", "screening", "measurement", "concentration", "urine", "rectal", "parenteral", "injection", "suppository", "gel" |
| mild topical corticosteroids | |
| "hydrocortisone cream", "hydrocortisone lotion", "hydrocortisone cutaneous", "hydrocortisone topical", "hydrocortisone ointment", "hydrocortisone acetate", "fluocinolone acetonide 25 microgram/1 gram" | "adverse reaction", "hydrocortisone butyrate", "eye ointment", "eye drops", "rectal", "suppository", "ear drops", "injection", "lidocaine", "pramoxine" |
| moderate topical corticosteroids | |
| "betamethasone cream", "betamethasone cutaneuous", "betamethasone lotion", "betamethasone ointment", "clobetasone topical", "clobetasone cutaneous", "fludroxycortide cutaneous", "alclometasone topical", "alclometasone cutaneous", "fluocinolone acetonide 62.5 microgram/g cutaneous" | "betamethasone dipropionate", "betamethasone 0.1%", "eye ointment", "calcipotriene" |
| potent topical corticosteroids | |
| "beclomethasone dipropionate cutaneous", "betamethasone valerate 0.1%", "betamethasone valerate 2.25mg", "betamethasone 0.1% foam", "fluticasone topical", "fluticasone cutaneous", "mometasone topical", "mometasone cutaneous", "hydrocortisone butyrate topical", "hydrocortisone butyrate cutaneous", "triamcinolone cutaneous", "triamcinolone topical", "triamcinolone cream", "triamcinolone ointment", "betamethasone dipropionate salicylic", "fluocinolone acetonide 250 microgram/g cutaneous", "\"diflucortolone valerate 1 mg/g cutaneous\"" | none |
| systemic immunosupressants | |
| "azathioprine", "mycophenolate", "cyclosporin", "ciclosporin", "cyclosporine", "azathioprin" | "poisoning", "disorder", "observable entity", "situation", "procedure", "embryopathy", "overdose", "adverse reaction", "induced by", "long-term current use", "caused by", "allergy", "nephrotoxicity", "level", "ophthalmic", "index", "measurement" |
| topical antibiotics | |
| "fusidic", "fusidate", "mupirocin" | "oral", "ophthalmic", "eye drops", "overdose", "poisoning", "adverse reaction", "allergy", "parenteral", "injection", "nasal", "infection" |
| topical calcineurin inhibitors | |
| "tacrolimus topical", "tacrolimus cutaneous", "pimecrolimus" | none |
| very potent topical corticosteroids | |
| "clobetasol", "\"diflucortolone valerate 3 milligram/1 gram\"" | disorder |

## eTable 3: Topical corticosteroid potency

| Name | Potency*^1^* | Compound | Generic*^2^* |
| --- | --- | --- | --- |
| Hydrocortisone 2.5% | mild |  | not found |
| Dioderm | mild |  | Hydrocortisone 0.1% cream |
| Mildison | mild |  | Hydrocortisone 1% cream |
| Synalar 1 in 10 dilution | mild |  | Fluocinolone acetonide 0.0025% cream |
| Canesten HC | mild | with antimicrobials | Hydrocortisone 1% / Clotrimazole 1% cream |
| Daktacort | mild | with antimicrobials | Hydrocortisone 1% / Miconazole 2% ointment |
| Econacort | mild | with antimicrobials | Econazole 1% / Hydrocortisone 1% cream |
| Fucidin H | mild | with antimicrobials | Hydrocortisone acetate 1% / Fusidic acid 2% cream |
| Hydrocortisone with chlorhexidine hydrochloride and nystatin | mild | with antimicrobials | not found |
| Terra-Cortril | mild | with antimicrobials | Oxytetracycline 3% / Hydrocortisone 1% ointment; Generic Terra-Cortril Nystatin cream |
| Timodine | mild | with antimicrobials | Generic Timodine cream |
| Betnovate-RD | moderate |  | Betamethasone valerate 0.025% cream |
| Eumovate | moderate |  | Clobetasone 0.05% cream |
| Haelan | moderate |  | Fludroxycortide 0.0125% cream; Fludroxycortide 4micrograms/square cm tape 7.5cm |
| Modrasone | moderate |  | Alclometasone 0.05% cream |
| Synalar 1 in 4 Dilution | moderate |  | Fluocinolone acetonide 0.00625% cream |
| Ultralanum Plain | moderate |  | Fluocortolone 0.25% / Fluocortolone hexanoate 0.25% ointment |
| Trimovate | moderate | with antimicrobials | Clobetasone 0.05% / Oxytetracycline 3% / Nystatin 100,000units/g cream |
| Alphaderm | moderate | with urea | Hydrocortisone 1% / Urea 10% cream |
| Beclometasone dipropionate 0.025% | potent |  | not found |
| Betamethasone valerate 0.1% | potent |  | Betamethasone valerate 0.1% cream |
| Betacap | potent |  | Betamethasone valerate 0.1% scalp application |
| Betesil | potent |  | Betamethasone valerate 2.25mg medicated plasters |
| Bettamousse | potent |  | Betamethasone 0.1% foam |
| Betnovate | potent |  | Betamethasone valerate 0.1% cream |
| Cutivate | potent |  | Fluticasone 0.05% cream; Fluticasone 0.005% ointment |
| Diprosone | potent |  | Betamethasone dipropionate 0.05% cream |
| Elocon | potent |  | Mometasone 0.1% cream |
| Hydrocortisone butyrate | potent |  | Hydrocortisone butyrate 0.1% cream |
| Locoid | potent |  | Hydrocortisone butyrate 0.1% cream |
| Locoid Crelo | potent |  | Hydrocortisone 0.1% topical emulsion |
| Metosyn | potent |  | Fluocinonide 0.05% cream |
| Mometasone furoate 0.1% | potent |  | Mometasone furoate 0.1% |
| Nerisone | potent |  | Diflucortolone 0.1% cream |
| Synalar | potent |  | Fluocinolone acetonide 0.025% ointment |
| Aureocort | potent | with antimicrobials | Triamcinolone acetonide 0.1% / Chlortetracycline 3.09% ointment |
| Betamethasone and clioquinol | potent | with antimicrobials | not found |
| Betamethasone and neomycin | potent | with antimicrobials | not found |
| Fucibet | potent | with antimicrobials | Betamethasone valerate 0.1% / Fusidic acid 2% cream |
| Lotriderm | potent | with antimicrobials | Betamethasone dipropionate 0.064% / Clotrimazole 1% cream |
| Synalar C | potent | with antimicrobials | Fluocinolone acetonide 0.025% / Clioquinol 3% ointment |
| Synalar N | potent | with antimicrobials | Fluocinolone acetonide 0.025% / Neomycin 0.5% cream |
| Diprosalic | potent | with salicylic acid | Betamethasone dipropionate 0.05% / Salicylic acid 3% ointment |
| Clarelux | very potent |  | Clobetasol 500micrograms/g foam |
| Dermovate | very potent |  | Clobetasol 0.05% cream |
| Etrivex | very potent |  | Clobetasol 500micrograms/g shampoo |
| Nerisone Forte | very potent |  | Diflucortolone 0.3% ointment |
| Clobetasol with neomycin and nystatin | very potent | with antimicrobials | not found |
| *^1^*Potency as per https://bnf.nice.org.uk/treatment-summaries/topical-corticosteroids/ | | | |
| *^2^*Virtual Medicinal Product (VMP) as per https://services.nhsbsa.nhs.uk/dmd-browser/ | | | |

## eTable 4: ALSPAC variables for flexural dermatitis presence and severity

|  | Presence | | Severity | |
| --- | --- | --- | --- | --- |
| timepoint (months) | name | label | name | Label^a^ |
| 6 | kb086 | CH had rash in joints & creases | kb087 | Severity of rash |
| 18 | kd085 | CH Had Rash in Joints Since Aged 6 MTHS | kd086 | Severity of Rash in Joints |
| 30 | kf110 | Child had rash in joints > 18 months | kf111 | Severity of child’s rash |
| 42 | kj100 | CH Had Dry Itchy Rash In Joints | kj101 | Severity of CHs Skin PROB |
| 57 | kl100 | A8a: Child had itchy, dry skin rash in joints since age 3 | kl101 | A8b: Severity of child's dry, itchy rash |
| 69 | kn1120 | A7a: Child had dry skin rash on joints and body creases in past 15 months | kn1121 | A7b: Severity of child's skin rash on joints and creases in past 15 months |
| 81 | kq090 | A7a: CH Had Itchy/Dry Skin Rash In Past Year | kq091 | A7b: How Bad Was CH Itchy/Dry Rash |
| 103 | ks1280 | A11a: Child had itchy, dry skin rash in the joints/creases of body | ks1281 | A11b: Extent of itchy dry skin rash |
| 128 | kv1111 | A8b: Child had itchy/dry rash in joints in past year | kv1112 | A8c: What is the severity of these problems |
| 140 | kw1280 | A16a: Child had any itchy, dry skin rash in the joints and creases of body in the past year | kw1281 | A16b: Severity of the rash |
| 166 | tb1111 | A8b: Child had itchy dry, skin rash in the last year | tb1112 | A8c: Severity of child's itchy dry, skin rash |
| ^a^parents could answer with very bad, quite bad, mild, no problem | | | | |

## eTable 5: Most common codes

| eczema | n |
| --- | --- |
| atopic dermatitis/eczema | 6461 |
| eczema nos | 3269 |
| infantile eczema | 2075 |
| atopic dermatitis and related | 1528 |
| flexural eczema | 536 |
| atopic_only_eczema | |
| atopic dermatitis/eczema | 6461 |
| atopic dermatitis and related | 1528 |
| atopic dermatitis nos | 411 |
| allergic_rhinitis | |
| hay fever - unspec allergen | 2243 |
| hay fever - pollens | 1325 |
| allergic rhinitis | 470 |
| allergic rhinitis nos | 168 |
| asthma |  |
| asthma | 16247 |
| asthma monitoring | 14228 |
| asthma monitored | 4848 |
| asthma annual review | 1864 |
| exercise induced asthma | 1831 |
| asthma_diagnosis | |
| asthma | 16247 |
| exercise induced asthma | 1831 |
| intrinsic asthma | 1686 |
| allergic asthma | 822 |
| acute exacerbation of asthma | 401 |
| eczema_infections | |
| impetigo | 4079 |
| molluscum contagiosum | 1252 |
| cellulitis/abscess - finger | 509 |
| cellulitis/abscess of toe | 348 |
| herpes simplex | 212 |
| folliculitis | |
| seborrhoea capitis | 234 |
| foodallergy | |
| peanut allergy | 87 |
| nut allergy | 68 |
| food allergy | 61 |
| insomnia | |
| [d]sleep disturbances | 145 |
| sleep disorders | 115 |
| c/o - insomnia | 95 |
| [d]insomnia nos | 68 |
| urticaria | |
| allergic urticaria | 172 |
| hives | 96 |
| adrenaline_pens | |
| epipen jr. 150micrograms/0.3ml (1 in 2,000) solution for injection auto-injectors (meda pharmaceuticals ltd) | 461 |
| epipen 300micrograms/0.3ml (1 in 1,000) solution for injection auto-injectors (meda pharmaceuticals ltd) | 271 |
| antibiotics | |
| amoxicillin 125mg/5ml oral suspension | 18745 |
| amoxicillin 250mg/5ml oral suspension | 2879 |
| amoxil 125mg/5ml syrup sucrose free (glaxosmithkline uk ltd) | 1861 |
| amoxicillin 250mg capsules | 1827 |
| amoxicillin 250mg/5ml oral suspension sugar free | 1753 |
| antihistamines | |
| cetirizine 10mg tablets | 2837 |
| loratadine 5mg/5ml oral solution | 2832 |
| cetirizine 1mg/ml oral solution sugar free | 1857 |
| chlorphenamine 2mg/5ml oral solution | 1665 |
| loratadine 10mg tablets | 1460 |
| asthma_inhalers | |
| salbutamol 100micrograms/dose inhaler cfc free | 12059 |
| beclometasone 100micrograms/dose inhaler | 8563 |
| becotide 50 inhaler (glaxosmithkline uk ltd) | 1725 |
| salbutamol 100micrograms/dose breath actuated inhaler cfc free | 1593 |
| salmeterol 25micrograms/dose inhaler | 1339 |
| insomnia_drugs | |
| phenergan 5mg/5ml elixir (sanofi) | 764 |
| hydroxyzine 10mg tablets | 53 |
| emollients | |
| oilatum emollient (glaxosmithkline consumer healthcare) | 8669 |
| e45 cream (forum health products ltd) | 4124 |
| diprobase cream (bayer plc) | 1479 |
| unguentum m cream (almirall ltd) | 771 |
| emulsiderm emollient (dermal laboratories ltd) | 382 |
| mild_topical_corticosteroids | |
| hydrocortisone 1% cream | 7234 |
| hydrocortisone 1% ointment | 2992 |
| hydrocortisone 0.5% cream | 1998 |
| timodine cream (alliance pharmaceuticals ltd) | 1667 |
| fucidin h cream (leo pharma) | 1460 |
| moderate_topical_corticosteroids | |
| eumovate 0.05% cream (glaxosmithkline uk ltd) | 1674 |
| eumovate 0.05% ointment (glaxosmithkline uk ltd) | 1335 |
| clobetasone 0.05% cream | 829 |
| clobetasone 0.05% ointment | 679 |
| alphaderm 1%/10% cream (alliance pharmaceuticals ltd) | 166 |
| potent_topical_corticosteroids | |
| fucibet cream (leo pharma) | 598 |
| betnovate rd 0.025% ointment (glaxosmithkline uk ltd) | 536 |
| betamethasone valerate 0.1% / fusidic acid 2% cream | 267 |
| betnovate rd 0.025% cream (glaxosmithkline uk ltd) | 248 |
| betnovate 0.1% cream (glaxosmithkline uk ltd) | 231 |
| oral_corticosteroids | |
| prednisolone 5mg soluble tablets | 1464 |
| prednisolone 5mg gastro-resistant tablets | 481 |
| prednisolone 5mg tablets | 407 |
| hydrocortisone 10mg tablets | 116 |
| betamethasone 500microgram soluble tablets sugar free | 55 |
| topical_antibiotics | |
| fucidin 20mg/g cream (leo pharma) | 1895 |
| fucidin h cream (leo pharma) | 1460 |
| fusidic acid 2% cream | 1064 |
| fucidin h ointment (leo pharma) | 407 |
| mupirocin 2% ointment | 298 |

## eTable 6: Comparing AD in EHRs to AD symptom reports in ALSPAC as the reference standard

| Definition of AD in EHRs | True positives^a^ | True negatives^a^ | False positives^a^ | False negatives^a^ | Sensitivity^a^ | Specificity^a^ |
| --- | --- | --- | --- | --- | --- | --- |
| >1 reports of AD symptoms in ALSPAC as the reference standard | | | | | | |
| AD diagnosis and prescription | 1425 | 3336 | 354 | 3713 | 0.28 | 0.90 |
| AD diagnosis or prescription | 3017 | 2538 | 1152 | 2121 | 0.59 | 0.69 |
| AD diagnosis | 1832 | 3171 | 519 | 3306 | 0.36 | 0.86 |
| >2 reports of AD symptoms in ALSPAC as the reference standard | | | | | | |
| AD diagnosis and prescription | 1162 | 4828 | 617 | 2221 | 0.34 | 0.89 |
| AD diagnosis or prescription | 2275 | 3551 | 1894 | 1108 | 0.67 | 0.65 |
| AD diagnosis | 1466 | 4560 | 885 | 1917 | 0.43 | 0.84 |
| ^a^True positives, true negatives, false positives, false negatives, sensitivity, and specificity when comparing AD in EHRs (using different definitions), to having >1 or >2 reports of AD symptoms in ALSPAC as the reference standard. | | | | | | |

## eTable 7: Proportions of people with AD in primary care, by subtype

|  | AD in primary care? | |  |
| --- | --- | --- | --- |
| AD definition in EHRs | yes | no | Proportion |
| **Severe-frequent** | | | |
| AD diagnosis and treatment | 237 | 119 | 67% |
| AD diagnosis or treatment | 327 | 29 | 92% |
| AD diagnosis | 269 | 87 | 76% |
| **Moderate-frequent** | | | |
| AD diagnosis and treatment | 352 | 364 | 49% |
| AD diagnosis or treatment | 600 | 116 | 84% |
| AD diagnosis | 433 | 283 | 60% |
| **Moderate-declining** | | | |
| AD diagnosis and treatment | 364 | 761 | 32% |
| AD diagnosis or treatment | 786 | 339 | 70% |
| AD diagnosis | 467 | 658 | 42% |
| **Mild-intermittent** | | | |
| AD diagnosis and treatment | 309 | 563 | 35% |
| AD diagnosis or treatment | 606 | 266 | 69% |
| AD diagnosis | 363 | 509 | 42% |
| **Unaffected/Rare** | | | |
| AD diagnosis and treatment | 948 | 4813 | 16% |
| AD diagnosis or treatment | 2881 | 2880 | 50% |
| AD diagnosis | 1284 | 4477 | 22% |
| Explanatory examples: Of those with Severe-Frequent AD subtype in ALSPAC, 76% ever had AD, 92% ever had AD or an AD treatment, and 67% ever had AD and an AD treatment in EHRs. Of those with unaffected/rare AD subtype in ALSPAC, 22% ever had AD, 50% ever had AD or an AD treatment, and 16% ever had AD and an AD treatment in EHRs. | | | |

## eTable 8: Confusion Matrix

|  | Truth | | | | |
| --- | --- | --- | --- | --- | --- |
| Prediction | 1 | 2 | 3 | 4 | 5 |
| 1 | 22 | 17 | <10 | <10 | <10 |
| 2 | 10 | 24 | 11 | <10 | 11 |
| 3 | <10 | 13 | <10 | <10 | <10 |
| 4 | <10 | <10 | <10 | <10 | <10 |
| 5 | 35 | 130 | 244 | 186 | 1437 |
| 1=Severe-Frequent; 2=Moderate-Frequent; 3=Moderate-Declining; 4=Mild-Intermittent; 5=Unaffected/Rare | | | | | |

## eTable 9: Metrics by definition of outcome variable and by predictor set used

| Predictor Set | ROC AUC | Accuracy | Sensitivity | Specificity |
| --- | --- | --- | --- | --- |
| Original Subtypes (n=6,622) | | | | |
| 1: Presence of AD prescription and diagnosis codes in 1-year windows | 0.65 | 0.68 | 0.29 | 0.83 |
| 2: Ever/never had code for a given disease/treatment | 0.63 | 0.66 | 0.28 | 0.82 |
| 3: How often had code for a given disease/treatment | 0.63 | 0.66 | 0.25 | 0.82 |
| 4: Age of first occurrence for a given disease/treatment | 0.64 | 0.66 | 0.27 | 0.83 |
| 5: Presence of code for a given disease/treatment in 1-year windows | 0.63 | 0.65 | 0.27 | 0.83 |
| 1 + 3 | 0.68 | 0.67 | 0.31 | 0.83 |
| 1+ 3 + 5 | 0.64 | 0.67 | 0.3 | 0.83 |
| Binary Subtypes (n=6,622) | | | | |
| 1: Presence of AD prescription and diagnosis codes in 1-year windows | 0.7 | 0.72 | 0.26 | 0.96 |
| 2: Ever/never had code for a given disease/treatment | 0.69 | 0.7 | 0.37 | 0.89 |
| 3: How often had code for a given disease/treatment | 0.71 | 0.71 | 0.27 | 0.94 |
| 4: Age of first occurrence for a given disease/treatment | 0.73 | 0.74 | 0.42 | 0.91 |
| 5: Presence of code for a given disease/treatment in 1-year windows | 0.7 | 0.72 | 0.29 | 0.96 |
| 1 + 3 | 0.72 | 0.73 | 0.26 | 0.97 |
| 1+ 3 + 5 | 0.71 | 0.74 | 0.33 | 0.95 |
| Three category Subtypes (n=6,622) | | | | |
| 1: Presence of AD prescription and diagnosis codes in 1-year windows | 0.69 | 0.7 | 0.42 | 0.74 |
| 2: Ever/never had code for a given disease/treatment | 0.7 | 0.7 | 0.45 | 0.75 |
| 3: How often had code for a given disease/treatment | 0.7 | 0.7 | 0.41 | 0.73 |
| 4: Age of first occurrence for a given disease/treatment | 0.71 | 0.69 | 0.44 | 0.75 |
| 5: Presence of code for a given disease/treatment in 1-year windows | 0.7 | 0.67 | 0.4 | 0.72 |
| 1 + 3 | 0.71 | 0.69 | 0.45 | 0.74 |
| 1+ 3 + 5 | 0.71 | 0.68 | 0.41 | 0.72 |
| Four category Subtypes (n=6,622) | | | | |
| 1: Presence of AD prescription and diagnosis codes in 1-year windows | 0.66 | 0.66 | 0.33 | 0.78 |
| 2: Ever/never had code for a given disease/treatment | 0.66 | 0.66 | 0.32 | 0.79 |
| 3: How often had code for a given disease/treatment | 0.67 | 0.67 | 0.32 | 0.79 |
| 4: Age of first occurrence for a given disease/treatment | 0.66 | 0.66 | 0.33 | 0.79 |
| 5: Presence of code for a given disease/treatment in 1-year windows | 0.66 | 0.69 | 0.35 | 0.8 |
| 1 + 3 | 0.68 | 0.68 | 0.37 | 0.79 |
| 1+ 3 + 5 | 0.67 | 0.68 | 0.36 | 0.79 |
| Orignial (with more complete EHRs) (n=4,232) | | | | |
| 1: Presence of AD prescription and diagnosis codes in 1-year windows | 0.66 | 0.66 | 0.29 | 0.83 |
| 2: Ever/never had code for a given disease/treatment | 0.63 | 0.65 | 0.28 | 0.82 |
| 3: How often had code for a given disease/treatment | 0.62 | 0.65 | 0.28 | 0.83 |
| 4: Age of first occurrence for a given disease/treatment | 0.64 | 0.63 | 0.27 | 0.83 |
| 5: Presence of code for a given disease/treatment in 1-year windows | 0.65 | 0.66 | 0.28 | 0.83 |
| 1 + 3 | 0.67 | 0.67 | 0.3 | 0.83 |
| 1+ 3 + 5 | 0.65 | 0.67 | 0.28 | 0.82 |
| Original (with complete ALSPAC follow-up) (n=657) | | | | |
| 1: Presence of AD prescription and diagnosis codes in 1-year windows | 0.72 | 0.31 | 0.24 | 0.81 |
| 2: Ever/never had code for a given disease/treatment | 0.61 | 0.31 | 0.22 | 0.81 |
| 3: How often had code for a given disease/treatment | 0.67 | 0.36 | 0.29 | 0.83 |
| 4: Age of first occurrence for a given disease/treatment | 0.63 | 0.3 | 0.23 | 0.81 |
| 5: Presence of code for a given disease/treatment in 1-year windows | 0.66 | 0.34 | 0.27 | 0.81 |
| 1 + 3 | 0.67 | 0.41 | 0.31 | 0.83 |
| 1+ 3 + 5 | 0.69 | 0.37 | 0.29 | 0.83 |
| Original (excluding Unaffected/Rare) (n=2,302) | | | | |
| 1: Presence of AD prescription and diagnosis codes in 1-year windows | 0.66 | 0.43 | 0.33 | 0.78 |
| 2: Ever/never had code for a given disease/treatment | 0.64 | 0.39 | 0.27 | 0.76 |
| 3: How often had code for a given disease/treatment | 0.67 | 0.41 | 0.32 | 0.77 |
| 4: Age of first occurrence for a given disease/treatment | 0.65 | 0.39 | 0.29 | 0.77 |
| 5: Presence of code for a given disease/treatment in 1-year windows | 0.68 | 0.43 | 0.34 | 0.78 |
| 1 + 3 | 0.68 | 0.42 | 0.35 | 0.78 |
| 1+ 3 + 5 | 0.67 | 0.42 | 0.35 | 0.78 |
| ^a^Outcomes:  2 categories (1: Unaffected/Rare; 2: Mild-Intermittent + Moderate-Declining + Moderate-Frequent + Severe-Frequent).  3 categories (1: Unaffected/Rare; 2: Mild-Intermittent + Moderate-Declining + Moderate-Frequent; 3: Severe-Frequent)  4 categories (1: Unaffected/Rare; 2 Mild-Intermittent + Moderate-Declining; 3: Moderate-Frequent; 4: Severe-Frequent)  Except for the 2 category outcome variable, ROC AUC is averaged using the method by Hand, Till (2001), and sensitivity and specificity are macro averaged. | | | | |

# TRIPOD check-list for Prediction Model Development

### Title and Abstract

1. Title: Identify the study as developing and/or validating a multivariable prediction model, the target population, and the outcome to be predicted. **Title**
2. Abstract: Provide a summary of objectives, study design, setting, participants, sample size, predictors, outcome, statistical analysis, results, and conclusions. **Abstract**

### Introduction

1. Background and objectives
   1. Explain the medical context (including whether diagnostic or prognostic) and rationale for developing or validating the multivariable prediction model, including references to existing models. **Introduction**
   2. Specify the objectives, including whether the study describes the development or validation of the model or both. **Objectives**

### Methods

1. Source of data
   1. Describe the study design or source of data (e.g., randomized trial, cohort, or registry data), separately for the development and validation data sets, if applicable. **Data sources**
   2. Specify the key study dates, including start of accrual; end of accrual; and, if applicable, end of follow-up. **Data sources**
2. Participants
   1. Specify key elements of the study setting (e.g., primary care, secondary care, general population) including number and location of centres. **Data sources**
   2. Describe eligibility criteria for participants. **Participants**
   3. (Give details of treatments received, if relevant.)
3. Outcome
   1. Clearly define the outcome that is predicted by the prediction model, including how and when assessed. **Outcomes**
   2. Report any actions to blind assessment of the outcome to be predicted. **Statistical analysis**
4. Predictors
   1. Clearly define all predictors used in developing or validating the multivariable prediction model, including how and when they were measured. **Predictors**
   2. Report any actions to blind assessment of predictors for the outcome and other predictors. **Statistical analysis**
5. Sample size: Explain how the study size was arrived at. **Participants**
6. Missing data: Describe how missing data were handled (e.g., complete-case analysis, single imputation, multiple imputation) with details of any imputation method. **Participants, Statistical analysis**
7. Statistical analysis methods
   1. Describe how predictors were handled in the analyses. **Statistical analysis**
   2. Specify type of model, all model-building procedures (including any predictor selection), and method for internal validation. **Statistical analysis**
   3. Specify all measures used to assess model performance and, if relevant, to compare multiple models. **Statistical analysis**
8. (Risk groups: Provide details on how risk groups were created, if done.)

### Results

1. Participants
   1. Describe the flow of participants through the study, including the number of participants with and without the outcome and, if applicable, a summary of the follow-up time. A diagram may be helpful. **Descriptive statistics and linkage**
   2. Describe the characteristics of the participants (basic demographics, clinical features, available predictors), including the number of participants with missing data for predictors and outcome. **Descriptive statistics and linkage**

14. Model development

- 1. Specify the number of participants and outcome events in each analysis. **Descriptive statistics and linkage; eTable: Metrics by Definition of outcome variable**
  2. (If done, report the unadjusted association between each candidate predictor and outcome.)

15. Model specification

- 1. Present the full prediction model to allow predictions for individuals (i.e., all regression coefficients, and model intercept or baseline survival at a given time point). **eTable: Full model specification**
  2. Explain how to the use the prediction model. **Discussion**

16. Model performance: Report performance measures (with CIs) for the prediction model. **Predicting ALSPAC subtypes from primary care records**

### Discussion

1. Limitations: Discuss any limitations of the study (such as nonrepresentative sample, few events per predictor, missing data). **Limitations**
2. Interpretation: Give an overall interpretation of the results, considering objectives, limitations, and results from similar studies, and other relevant evidence. **Conclusions**
3. Implications: Discuss the potential clinical use of the model and implications for future research. **Conclusions**

### Other information

1. Supplementary information: Provide information about the availability of supplementary resources, such as study protocol, Web calculator, and data sets. **Appendix**
2. Funding: Give the source of funding and the role of the funders for the present study. **Acknowledgements**
